# Mutagen-Specific Mutation Signature Determines Global microRNA Binding

**DOI:** 10.1371/journal.pone.0027400

**Published:** 2011-11-09

**Authors:** Eyal Greenberg, Gideon Rechavi, Ninette Amariglio, Oz Solomon, Jacob Schachter, Gal Markel, Eran Eyal

**Affiliations:** 1 Ella Institute of Melanoma, Sheba Medical Center, Ramat-Gan, Israel; 2 Department of Clinical Microbiology and Immunology, Sackler Faculty of Medicine, Tel Aviv University, Tel Aviv, Israel; 3 Cancer Research Center, Sheba Medical Center, Ramat-Gan, Israel; 4 Sackler Faculty of Medicine, Tel Aviv University, Tel Aviv, Israel; 5 Faculty of Life Sciences, Bar-Ilan University, Ramat Gan, Israel; 6 Talpiot Medical Leadership Program, Sheba Medical Center, Ramat-Gan, Israel; University of Pennsylvania School of Medicine, United States of America

## Abstract

Micro-RNAs (miRNAs) are small non-coding RNAs that regulate gene products at the post-transcriptional level. It is thought that loss of cell regulation by miRNAs supports cancer development. Based on whole genome sequencing of a melanoma tumor, we predict, using three different computational algorithms, that the melanoma somatic mutations globally reduce binding of miRNAs to the mutated 3′UTRs. This phenomenon reflects the nature of the characteristic UV-induced mutation, C-to-T. Furthermore, we show that seed regions are enriched with Guanine, thus rendering miRNAs prone to reduced binding to UV-mutated 3′UTRs. Accordingly, mutation patterns in non UV-induced malignancies e.g. lung cancer and leukemia do not yield similar predictions. It is suggested that UV-induced disruption of miRNA-mediated gene regulation plays a carcinogenic role. Remarkably, dark-skinned populations have significantly higher GC content in 3′UTR SNPs than light-skinned populations, which implies on evolutionary pressure to preserve regulation by trans-acting oligonucleotides under conditions with excess UV radiation.

## Introduction

Melanoma is one of the main life-threatening malignancies of our era, accounting for 75% of skin cancer–related deaths worldwide [1]. Transformation and development of metastasis require stepwise acquisition of genetic and functional alterations [2], [3]. The roles of epigenetic and post-transcriptional mechanisms in melanoma are in the focus of recent studies [4], [5].

miRNAs are small, non-coding, 19–22 nucleotide long RNA strands, which function as specific epigenetic regulators of gene expression by inhibiting protein translation, leading mRNA to degradation, or both [6], [7]. The “seed” region, located between nucleotides 2 to 8 of the mature miRNA, binds to complementary regions in the 3′ un-translated region (3′UTR) of target mRNA to direct post-transcriptional repression. To date, nearly 1000 human miRNAs have been identified [8], and those are thought to regulate more than 50% of human genes [9]. Not surprisingly, their expression pattern is frequently perturbed in developmental diseases and cancer, which can directly exert phenotypic effects [10], [11], [12], [13], [14], [15]. In melanoma, several studies pointed on individual miRNAs whose expression level is likely to be related to formation and development of the cancer [16], [17], [18] or to the aggressive phenotype of melanoma cells [19]. Exemplar miRNAs include the inhibitory miRNAs miR-34a [19], miR-193b [20], let-7a [21], and miR-211 [22], [23], while miR-182 [24] and miR-221/222 [22] were shown to stimulate metastatic potential of melanoma cells.

Over recent years it was suggested that miRNAs are globally down regulated in human cancers [7]. Supporting this claim, it was shown that impaired processing of miRNAs promotes cellular transformation and tumorogenesis [25]. This trend was demonstrated in lung-cancer [26] and melanoma lymph node metastasis [16], in which more miRNAs were down-regulated than up-regulated. Cancer cells also exhibit frequent genomic alterations in regions of miRNA genes, reflected mainly as copy number variations [27]. These alterations are highly specific to the cancer type. In melanoma, 86% of the 283 examined miRNAs exhibited copy number variations, more than any other tested cancer, with 198 gains and 235 losses. Among those, 83 gains and 160 losses were specific to melanoma and explicitly associated with transcriptional expression, meaning that the overall activity of miRNAs is expected to be reduced in melanoma [27]. Noteworthy, this general trend may be cancer-specific, as global up-regulation of miRNA expression was observed in other solid malignancies [15], [28]. Current research therefore focuses on exploring differentially expressed miRNAs in cancer cells versus their normal counterparts and to relate these changes to cancer development and progression. There is still, however, a major gap in the understanding of how somatic alterations affect global cell regulation by expressed miRNAs, for example by altering their binding sites.

Recent studies employed enhanced sequencing technologies to obtain complete maps of genomic variants detected in tumors and compare them to normal tissues. These studies detected single nucleotide variants (SNV) [29] and more extensive genomic rearrangements in breast cancer [30], somatic mutations in a small cell lung cancer [31], acute myeloid leukemia (AML) [32], [33] and mesothelioma [34]. Typically, 20,000–30,000 genomic somatic mutations were found in each tumor genome, including hundreds within known genes, of which almost half within UTRs. It is still very difficult to identify the minority of mutations responsible directly to cancer formation (“driver” mutations). These studies emphasized that different cancers are characterized by different types of DNA aberrations, which are related to the mutagenic process. In melanoma, typical UV-induced single base substitutions account to more than 80% of all substitutions [31], [35] and primarily include C-to-T/A-to-G [36], [37], [38]. CC-to-TT is the prominent dinucleotide substitution [31]. The unique composition of UV-induced somatic mutations mandates examination of its effect on global cellular processes, such as global miRNA binding.

Here we show using three different computational tools that the UV-induced pattern of somatic mutations in melanoma reduces the general binding of miRNAs to mutated 3′UTRs. To the extent of our knowledge, this is the first study to predict disruption of miRNA-mediated regulation of gene expression due to a carcinogen. The implications of these observations are discussed, especially in light of a striking difference identified in nucleotide composition of SNP sites within the 3′UTRs between light- and dark-skinned populations.

## Results

### Mutations in the 3′UTR found in melanoma globally reduce miRNA binding

SNV analysis between melanoma cells and normal melanocytes revealed a ∼1∶1 ratio between those found within coding sequences and untranslated regions (UTRs) of known genes [31]. We hypothesized that miRNA binding to the mutated UTRs will be altered as compared to the normal UTRs.

**Figure 1:**
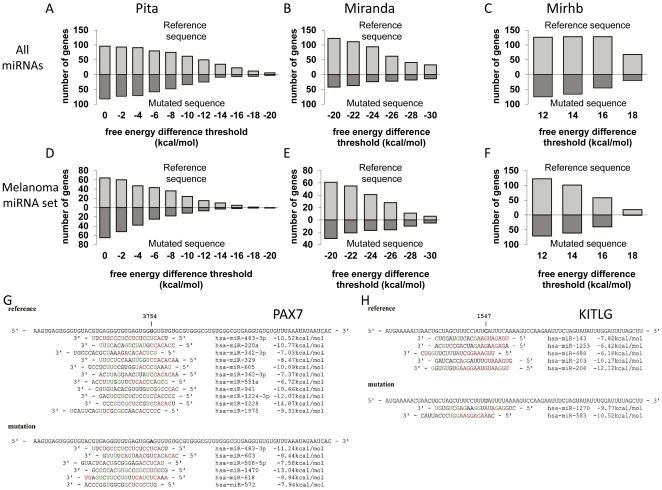
Global changes in miRNA binding due to 3′UTR mutations in melanoma and illustrative examples. The upper part of subplots A–F depicts the numbers of genes predicted to have more miRNA binding sites in the wild type sequence. The lower part of the subplots depicts the total numbers of genes predicted to have more miRNA binding sites in mutated version of the genes. Each program was applied several times using different threshold for miRNA binding (x-axis). **A.** Results according to pita [39] (x-axis units are ΔΔG threshold to define miRNA binding in each run). **B.** Results according to miRanda [54] (x-axis units are ΔΔG threshold to define miRNA binding in each run). **C.** Results according to mirhb (x-axis units are the numbers of satisfied hydrogen bonds in the seed region define miRNA binding in each run). **D.** Results according to pita but only based on miRNAs expressed in melanoma [16]. **E**. Results according to miRanda only based on miRNAs expressed in melanoma. **F.** Results according to mirhb only based on miRNAs expressed in melanoma. **G.** Changes in miRNA binding in paired-box 7 (PAX7) caused by melanoma somatic mutations. Red color indicates a perfect base pairing whereas green color indicates wobble U:G base paring. In each example the upper panel shows all miRNAs predicted by pita [39] to bind to the reference (wild type) sequence and the lower the miRNAs predicted to bind to mutated sequence along with their respective predicted ΔΔG binding free energy. **H.** Changes in miRNA binding in KIT ligand (KITLG).

All 207 point mutations discovered within 3′UTRs of known genes were analyzed by prediction tools for global miRNA binding to the 3′UTRs. Strikingly, Pita program [39] predicted a highly significant global miRNA binding preference to the reference genomic sequence over the mutated sequence (p = 2.5×10^−6^) in the melanoma patient genome, independently of the exact ΔΔG threshold chosen to define binding (Figure 1A). These results were further ratified (p = 0.0057) with a second prediction tool, miRanda, also under a wide range of ΔΔG threshold values (Figure 1B). Moreover, Mirhb, an in-house code to detect target sites for miRNAs based on defined number of satisfied hydrogen bonds, showed the same phenomenon (p = 0.0046) over a wide range of number of hydrogen bond satisfaction (Figure 1C). Importantly, similar results with all three miRNA target prediction programs were also obtained (p = 0.0028, 0.035, 0.033 with pita, miRanda and Mirhb respectively) upon re-calculation for a selected set of 167 miRNAs, which was defined as “Melanoma miRNA set” by Caramuta et al [16] (Figure 1, D–F).

This phenomenon is exemplified here for two private cases: PAX7, a homeodomain transcription factor that plays important roles during fetal development and cancer growth [40] (Figure 1G), and KITLG, a ligand of the KIT tyrosine-kinase receptor, which acts in cellular development [41] (Figure 1H). Overexpression of both genes has been implicated in melanoma [42], [43] and both received high scores by the prediction programs, with PAX7 ranked first according to consensus prediction of the three algorithms (Table S1). These examples well represent the general trend, in which putative miRNA binding sites covering the somatic mutation sites tend to vanish due to the mutations rather than be created.

### Global reduction in miRNA binding is specific to melanoma as a consequence of the typical UV-induced mutations

**Figure 2:**
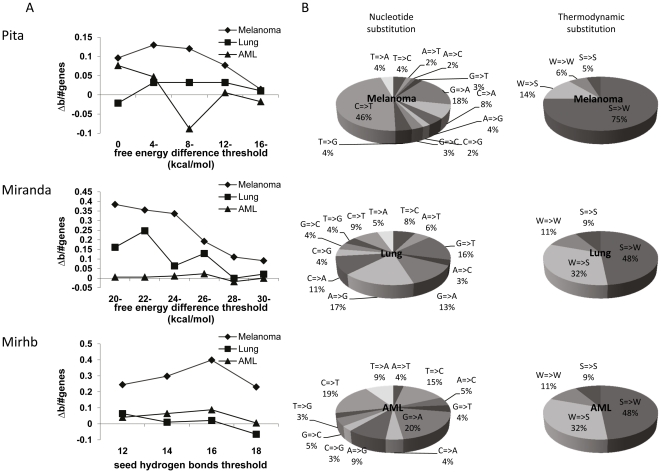
Global changes in miRNA binding due to 3′UTR mutations in melanoma, lung cancer and AML and distribution of mutation types. **A.** Shown are normalized differences between the total number of genes predicted to bind more miRNAs in the wild type sequences and the total number of genes predicted to bind more miRNAs in the mutated sequence (Δb over the total number of 3′UTR mutated genes) as predicted by the different programs. Each program was applied several times using different thresholds for miRNA binding (x-axis). **B.** Distribution of mutation types in different cancer genomes [31], [32], [39]. The left pie charts show the row distribution of mutations while the right charts show the distribution of mutations classified as Strong (S) or Weak (W) based on the number of hydrogen bonds in the base pairing (S = 3, W = 2).

The effect of mutations in the 3′UTRs on global miRNA binding in melanoma was compared to two other recently published complete cancer genomes, small cell lung cancer [31] and AML [32]. According to all three programs, the percentage of genes with predicted reduced miRNA binding due to the mutations was significantly larger in melanoma as compared to the other malignancies (Figure 2A). This general trend was observed over a wide range of any chosen parameter applied to define miRNA binding (Figure 2A). It is implied, therefore, that global reduction in miRNA binding is characteristic of melanoma. Indeed, an SNV-based analysis of the types of nucleotide substitutions verified that the melanoma set of mutations is unique by a very high frequency of C-to-T/G-to-A transitions (64.5% of all substitutions, Figure 2B left column), which are known to be induced by UV radiation [31], [36], [37], [38]. The second most common substitution in 3′UTR was found to be C-to-A/G-to-T, in agreement with the mutation frequencies for the entire genome [31]. Taken together, the two leading types of mutations in melanoma are of type Strong-to-Weak in terms of thermodynamic hybridization stability. Moreover, the melanoma mutation profile is dramatically skewed, as 75% of all substitutions are of Strong-to-Weak, while the reciprocal Weak-to-Strong substitutions account for only 13.9% (Figure 2B, right column). The mutation profiles of lung cancer or AML were more balanced, with 47–48% of Strong-to-Weak and ∼32% of Weak-to-Strong substitutions (Figure 2B, right column). Such mutations are expected to reduce the thermodynamic stability of any cis- or trans-hybridizations.

**Figure 3:**
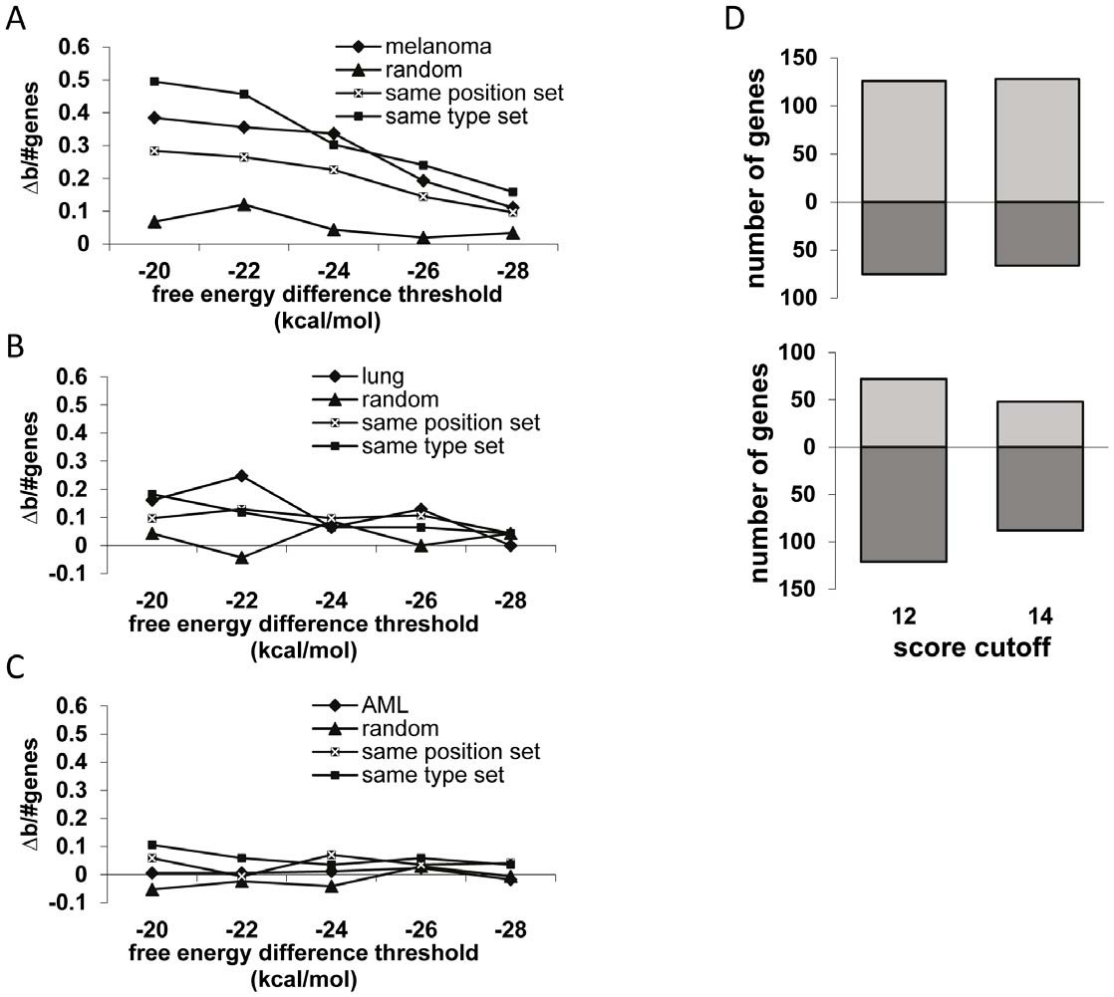
Global changes in miRNA binding due to 3′UTR mutations vs.*in silico* mutations. **A**. Global changes in miRNA binding due to 3′UTR mutations in real melanoma mutations compared to sets with random *in-silico* mutations. Shown are normalized differences between the numbers of genes predicted to bind more miRNAs in the wild type sequences and the numbers of genes predicted to bind more miRNAs in the mutated sequence (Δb over number of 3′UTR mutated genes). Predictions were made using miranda. See text for description of the different random sets. **B.** Global changes in miRNA binding due to 3′UTR mutations in small cell lung cancer mutations compared to sets with random *in-silico* mutations. **C.** Global changes in miRNA binding due to 3′UTR mutations in AML mutations compared to sets with random *in-silico* mutations. **D.** Global changes in miRNA binding when GC base paring is thermodynamically favored (upper panel) and when artificially set to be equal to AU pairing (lower panel). The upper part of each panel depicts the numbers of genes predicted to have more miRNA binding sites in the wild type sequence. The lower part of each panel depicts the total numbers of genes predicted to have more miRNA binding sites in the mutated version of the genes. This figure emphasizes that the preferred miRNA binding in the wild-type sequences over the mutants is not due to ruining of particular sequence motifs, but due to the enhanced thermo stability of the CG base pairing.

The reduced global binding in melanoma was next studied with Miranda in random sets of point mutations in the same 3′UTRs, in order to determine whether it is restricted to the mutation sites or results from the characteristic melanoma mutation profile type (Figure 3A). There was clearly no reduced binding in two sets, in which the position of the mutation and its type were both randomly chosen. To examine the importance of the type of substitution we constructed a set with *in silico* mutations of the same type as the originally observed mutations in randomly located sites along the 3′UTR (“same type set”). Remarkably, this set demonstrated similar overall results to the original set of the real mutations, confirming that the type of substitutions accounts for the reduced miRNA binding, with little or no contribution to their exact location. A third random set of which the position of the mutation was preserved but the type was randomly changed (“same position set”) demonstrated an intermediate effect (Figure 3A). It should be mentioned that due to the composition of somatic mutations in melanoma this set is biased in its composition, which could explain the intermediate effect. Equivalent analysis for the other cancers, such as lung cancer (Figure 3B) or AML (Figure 3C) reveals less apparent differences in global miRNA binding between the real mutated sequences and the sets with different types of *in silico* mutations, as described above. This observation is consistent with the hypothesis that the unique biased composition of melanoma somatic mutations accounts for the reduced miRNA binding phenomenon.

We noticed that there is a clear enrichment (p<10^-16^) in GC content (mainly G) in the nucleotide composition of miRNAs (49.3%) and within the seed regions (50.9%), as compared to the GC content of the 3′UTRs (42.4%) (Figure S1). This supports the notion on the importance of the thermodynamic advantage of GC pairs in miRNA binding. We used Mirhb to verify that the weaker binding of miRNAs to 3′UTRs is due to the special composition of mutations and not due to modifications of entire sequence motifs. In order to test this hypothesis, we set the parameters such that GC pairs contribute equally to the binding as AT pairs. Importantly, following this modification we obtained completely different results, with more genes now showing preferred miRNA binding to the mutated sequences (Figure 3D). Taken together, the significant role played by GC pairs in miRNA binding and their relative abundance within miRNAs and their seed regions, suggests that the GC nucleotides in the 3′UTRs have a greater contribution for overall miRNA binding, despite their scarcity compared to AT (Figure S1). Moreover, as the G content in miRNAs and seed regions is higher by ∼20% than the C content (Figure S1), it is logical to assume that the complementary C nucleotides in the 3′UTRs have thus a greater overall significance than G nucleotides. These observations are in perfect agreement with results demonstrating that global miRNA binding is disrupted only by the melanoma mutations (Figure 2A), which commonly target C nucleotides (Figure 2B). There were no significant differences in GC content in the 3′UTRs of melanoma, AML and lung cancer mutated genes (Figure S1), which further supports the role of mutation type and not the specific local sequence context in controlling miRNA binding.

**Figure 4:**
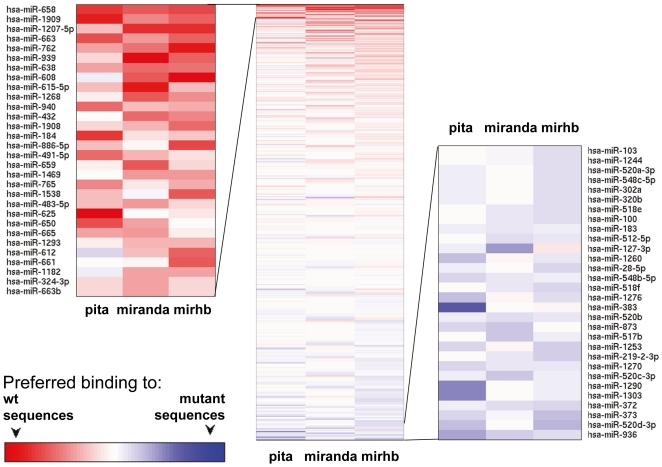
miRNAs sorted according to the difference between the number of 3′UTR miRNA binding sites predicted in the wild type sequences and the number of binding sites predicted in the mutated genes (Δb). Zoom-in is given for the top 30 miRNAs at both ends of the sorted list.

It is of interest to characterize the type of miRNAs most affected by the melanoma mutations, as this could shed further light on the underlying molecular mechanisms. We therefore sorted all miRNAs according to their differential binding to the mutated 3′UTRs, by a consensus prediction using the sum of the ranks for each program separately (Figure 4 and Table S2). Literature review revealed enrichment for down-regulated or tumor-suppressive properties among the top 20 miRNAs in the list (most reduced binding). Review of the bottom 20 miRNAs in the list (most enhanced binding) revealed enrichment for up-regulation or oncogenic properties (Table S2). Remarkably, target analysis with bioinformatics tools Toppgene [44] and miRror [45] of the top 20 miRNA highlighted biological processes contributing to oncogenic properties such as cell morphogenesis, development and actin cytoskeleton organization (Table S3). In marked contrast, target analysis of the bottom 20 miRNAs highlighted biological processes contributing to tumor suppressive properties, such as apoptosis and regulation of cell death (Table S3).

### Decreased miRNA binding as a possible evolutionary pressure

**Figure 5:**
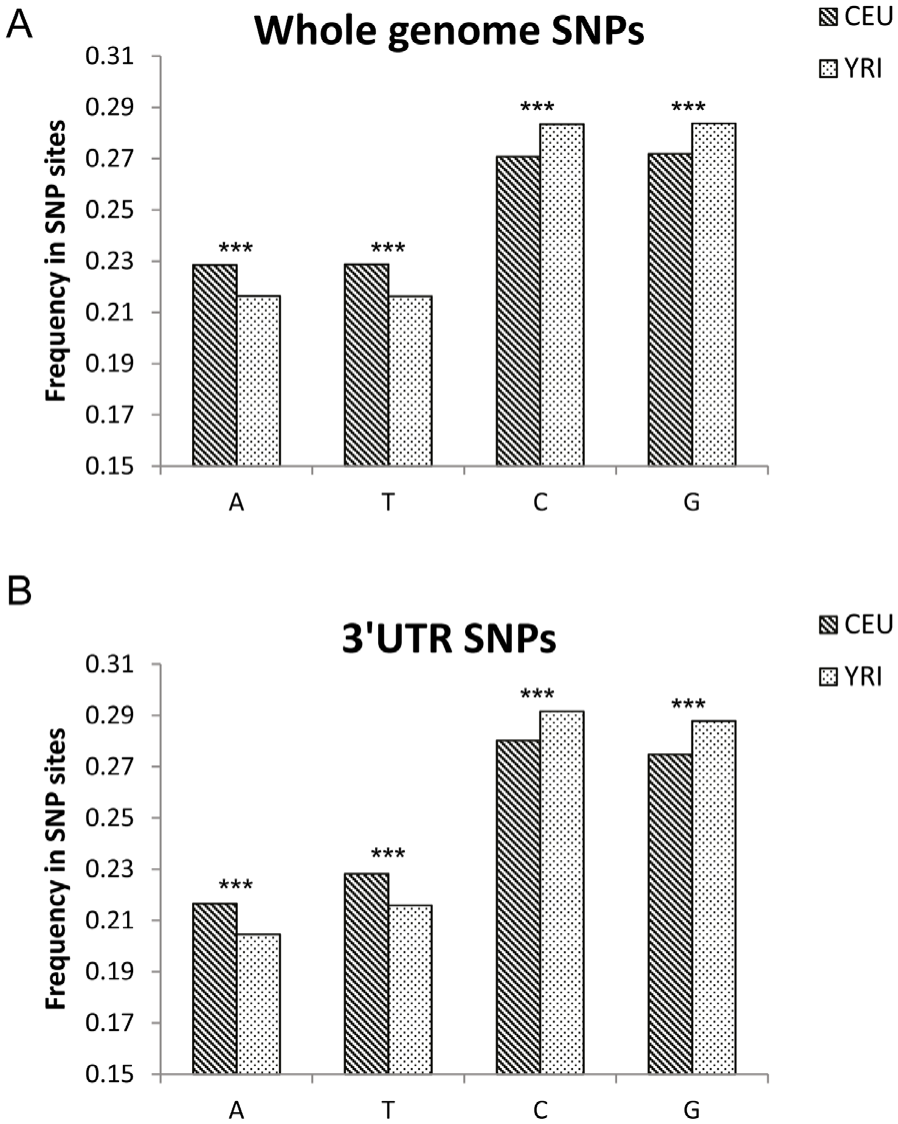
Base frequencies at SNP sites among Utah residents having European ancestry (CEU) and among Yoruba people from Nigeria, Africa (YRI). SNP frequencies were calculated based on the 1000 genomes project data. **A.** Whole genome SNPs analysis **B.** 3′UTR SNPs analysis.

It is known that dark-skinned populations have a significantly reduced risk to develop cutaneous melanoma [46]. While the molecular explanation is not fully understood, it is thought to be related to the more intense pigmentation, which protects against UV radiation, the main melanoma-inducing carcinogen. However, additional evolutionary adaptations that protect human populations at geographical regions characterized by excess sunlight may exist. We showed that the Strong-to-Weak mutations can potentially affect miRNA binding (Figure 3) and thus potentially alter normal regulation of the cell. We therefore speculated that significant natural differences in nucleic acid compositions might distinguish dark- and white-skinned populations. The GC (Strong) or AT (Weak) composition of SNP sites in genotyping database of Caucasians (Utah, originating from central Europe) and Sub-Saharan Africans (Nigeria) available at the 1000 genomes project [47], [48] was analyzed and compared. In both populations, there was clearly higher GC content over AT in SNP sites in the entire genome (Figure 5A), with a particular high ratio in 3′UTRs (Figure 5B and Table 1). However, a remarkable and significant difference (p<10^-16^) in the GC/AT ratio was noted between the two populations, with the ratio being greater in the Sub-Saharan population compared to the central European population. To exclude artifacts due to low coverage of SNP sites, we repeated the analysis only with SNPs with greater coverage and obtained essentially the same trend with even more apparent differences even at more stringent coverage thresholds (Table 1). Similar results were obtained when only compositions of SNPs reported in both populations (Table 1) were considered. Collectively, these observations could suggest higher GC content due to selective pressure against Strong-to-Weak mutations which may cause decreased miRNAs binding, gene dysregulation and ultimately transformation. Moreover, it proves that the GC enrichment in polymorphic sites happened in ancestral sites as well as in more recent sites acquired after the migration of the *Homo sapiens* from Africa some 100,000 years ago. We also examined exclusively the composition of the SNPs that fall within predicted miRNAs target sites. Strikingly, a large and significant (p = 10^-6^) difference was observed between the GC compositions of SNPs at target sites of miRNAs known to be expressed in Melanoma and the GC content of SNPs falling at target sites of other miRNAs (Table 1). The same trend was evident for both populations but was more apparent among the Africans.

**Table 1:** Nucleotide frequency at SNP sites based on the 1000 genomes project.

		YRI				CEU				p-value
		#SNPs	#SNP calls	%GC	%AT	#SNPs	#SNP calls	%GC	%AT	
DP^1^ = 5	genome	10546163	359168638	56.72	43.28	7722361	424769524	54.27	45.73	<E-16
	3′UTR	162148	5782684	57.95	42.05	118338	6542524	55.51	44.49	<E-16
	Melanoma miRNA targets	259	8472	56.95	43.05	187	10820	54.82	45.18	<E-16
	Other miRNA targets	1449	46080	51.41	48.59	1079	61934	51.09	48.81	0.3
DP = 8	genome	9701436	91323858	58.02	41.98	7667681	153696724	54.32	45.58	<E-16
	3′UTR	151938	1556216	59.11	40.89	117398	2434198	55.42	44.58	<E-16
	Melanoma miRNA targets	241	2070	58.50	41.50	186	4006	55.47	44.53	<E-16
	Other miRNA targets	1287	10974	53.06	46.94	1076	23082	51.30	48.70	0.0032
DP = 11	genome	5098112	18765018	59.29	40.71	6894889	49433786	54.43	45.57	<E-16
	3′UTR	87210	344426	60.29	39.79	105966	818104	55.48	44.52	<E-16
	Melanoma miRNA targets	103	358	60.34	39.66	171	1356	53.76	46.24	0.0977
	Other miRNA targets	628	2238	55.41	44.59	987	7640	51.75	48.25	0.0023
CommonSNPs^2^, DP = 5	genome	4955571	165131624	54.61	45.39	4959997	273988408	52.84	47.16	<E-16
	3′UTR	73131	2559830	55.57	44.43	73192	4061996	53.68	46.32	<E-16

1. Minimal deepness of coverage for each site counted.

2. SNPs appear in both populations.

## Discussion

Recent massive parallel cancer genomic sequencing studies are expected to be the tip of the iceberg, considering the need to detect specific functional genomic variants among tens of thousands of differential SNVs distinguishing tumors from healthy tissues. The ∼1:1 ratio between mutations in coding exons and UTRs [31] highlighted the potential causative relevance of UTR mutations, such as by altering miRNA binding sites.

UV radiation is the dominant mutagen involved in many cases of melanoma-genesis. Therefore, melanoma cells are expected to bear many Strong-to-Weak mutations due to the typical UV-induced DNA damage. This was indeed evident in the first complete genome of melanoma tumor [31] (Figure 2) and was further corroborated by whole exome sequencing of 14 matched normal and metastatic melanoma DNA samples [38]. Using various miRNA target prediction programs applying conceptually different algorithms, we show that these mutations are predicted to reduce overall miRNAs binding to 3′UTRs (Figure 1). The reduced binding is melanoma-specific (Figure 2), as it is mainly a consequence of the type of mutation rather than its exact position (Figure 3). It is therefore expected to be a general phenomenon, which would be valid for every set of mutations preserving the mutation frequencies observed in melanoma. We are well aware that miRNA target prediction level using today′s software may still be inaccurate. Nevertheless, three different algorithms and different binding thresholds demonstrated consistent results. We are therefore carefully drawing the global conclusions, which should not be biased or affected by incorrect prediction of particular miRNAs binding sites.

Previous studies reported on down-regulation of miRNA expression in several cancers [13] and that generic impairment in miRNA processing promotes cellular transformation and tumorigenesis [25]. Transcripts in cancer cells also exhibit shorter versions of 3′UTR which may cause loss of miRNA-mediated repression [49]. Our study is the first to address the function of expressed miRNAs from the aspect of target gene binding. The presented results imply that somatic mutations in melanoma disrupt miRNA-mediated regulation of many genes, which is consistent with the notions of reduced miRNA-mediated regulation in cancer. The studies claim for global up regulation of miRNAs in cancer [15], [28], could reflect tissue specific physiology, or in other cases an endogenous cellular compensation mechanism for the reduced binding, in an attempt to maintain regulation of gene expression. Here we attempted to analyze global functional trends regulated by the affected microRNAs (Figure 4 and Tables S2–3). miRNAs were ranked and clustered according to their calculated ability to bind wild type or mutant sequences. It seems that this ranking reflects some functional properties, however it should be considered with cautious as it is based on a single melanoma genome. Future sequencing of many whole melanoma genomes will allow accurate evaluation of the types of most affected miRNAs and their potential role in cancer development.

Usually, mutations detected in cancerous tissues are described as “passenger” or “driver” mutations, with the vast majority being “passengers” [37]. Random mutations may hit selected tumor suppressors or oncogenes and could lead, in individual genes level, to cancer formation and progression. Here we suggest a new intermediate class of “dormant driver” mutations. Such mutations bear a modest functional effect, such as reduction of few miRNA binding to their target genes, which may not manifest phenotypically on the individual gene level. On the system level, however, when accumulation of such mutations in many genes reaches a critical point, a significant global perturbation in expression pattern of many genes may shift the system off-balance, severe cell dysregulation and potentially carcinogenesis might ensue. This hypothesis is in line with recently published study demonstrated the role of cryptic genetic variation in the evolution of RNA enzymes [50]. Indeed, in many instances cancer development cannot be fully explained based on combination of mutations in known oncogenes and tumor suppressors.

UV is a mutagen, which is known to exert its carcinogenic effect by introducing random “driver” and “passenger” mutations into coding and regulatory elements of individual genes [31]. Generation of “dormant driver” mutations comprises a new, UV-specific, mechanism of carcinogenesis. This UV-unique mechanism is probably completely independent from the conventional mutagenic effects of UV. Interestingly, both the nature of miRNA binding, which is highly dependent on GC pairs (Figure 3), and the presumed greater contribution of 3’UTR C nucleotides for miRNA binding, render the miRNA-binding sites highly susceptible to UV mutagenesis, because it causes mainly C-to-T mutations. Furthermore, it is expected to be applicable also in other UV-facilitated non-melanoma malignancies, such as squamous cell and basal cell carcinomas. This provides further mechanistic explanation for the role of UV as a major cause for the most common human malignancy, skin cancer [51].

Finally, we show that there is a surprising, highly significant, difference in the GC/AU ratio in the 3’UTRs between dark- and light-skinned human populations (Figure 5B). The importance of GC nucleotides and their thermodynamic advantage in conferring thermodynamic stability to DNA structures in response to UV-induced damage was suggested previously [52]. Here we suggest a novel role for GC nucleotides as facilitators of miRNA binding and thereby global cell regulation. It is tempting to speculate that this might be an evolutionary strategy to minimize UV radiation effects and reduce risk for development of skin cancer. The observed differences in GC content between SNPs falling within predicted target sites of miRNAs highly expressed in melanoma and target sites of other miRNAs strongly supports this speculation. The context or role of this observation in differential melanoma morbidity rates between dark- and light-skinned human populations remains to be elucidated.

## Materials and Methods

### Datasets of cancer mutations

Datasets of SNVs obtained using massively parallel genomic sequencing of cancer patients were obtained for melanoma [31], small cell lung cancer [31] and AML [32]. The first two sets were obtained from the supplementary materials of the publications. The complete list of the AML mutations was obtained from dbGAP of NCBI [53]. The datasets and the reference coordinates were based on version NCBI36 (HG18) of the human genome.

### MiRNA target predictions

Pita [39] and Miranda [54] were applied for prediction of miRNA targets. Miranda uses complementary sequence information for prediction. Pita applies additional criteria, including accessibility of the target molecules to trans-acting factors which improved prediction accuracy. We ran each of the programs using different ΔΔG thresholds to define miRNA binding.

For global analysis of miRNA binding we defined Δb as the number of genes with more miRNAs bound to the wild type sequence minus number of genes with more miRNAs bound to the mutated sequence.

For global analysis of miRNA targets and their overlap with SNP sites we used pre-calculated Pita targets available in the web site: 

(http://genie.weizmann.ac.il/pubs/mir07/mir07_data.html)

### Mirhb - target prediction program based on satisfied hydrogen bonds

A new in-house tool for miRNA target prediction, Mirhb, has been applied. Mirhb searches for miRNA targets according to the number of satisfied hydrogen bonds rather then percent identity or number of base mismatches. Due to its simple scoring scheme the program is extremely fast and can be used to check binding of many miRNAs vs. many UTRs, a utility not provided by most existing miRNA target prediction programs. The mirhb script, written in Perl is available at http://sheba-cancer.org.il/software/mirhb.

### MiRNA database

We used version 9.09 (September 2009) of mirBase [55] which holds 718 highly confident miRNAs.

### Gene annotations

Annotations and coordinates of genes were taken from the UCSC genome browser web site using the Table Browser tool [48].

### 1000 genomes project data

Files with genotyping of 100 individuals of Utah residents with Northern Western European ancestry (CEU) and of 100 individuals of the Yoruba in Ibadan, Nigeria (YRI) were downloaded from the pilot1 section in the 1000 genomes project site (http://www.1000genomes.org/) [47], [48]. These files list the genotyping of all sequenced individuals in each SNP position with supportive data on quality and coverage deepness.

### MiRNA expression data in melanoma

GEO experiment GSE19387 which holds the data from the work of Caramuta et al. [16] was used to define miRNAs changing expression in melanoma.

We used data from Hoek et al. [56] (GEO GSE4570) to detect genes which undergo expression change in melanoma cells.

### Prediction of commonly regulated targets

For prediction of commonly regulated targets for selected set of miRNAs, the mirror [45] algorithm was applied using p-value<0.05 as threshold.

### Prediction of biological processes

For prediction of biological processes Toppgene [44] algorithm was applied with p-value<0.05 as threshold.

### Statistical analysis

To compare GC content of entire UTRs and miRNA binding regions and GC content at SNP sites between different populations, chi-square test was applied. To examine the significance of the difference between genes having more miRNAs bound to the reference sequence and those with more miRNAs bound to the mutated sequences at different binging thresholds we used paired t-test.

## Supporting Information

Table S1 **3′UTR mutations in a melanoma genome.** The genes are sorted by consensus Δb value calculated by the different programs.
(DOC)

Table S2 **Prediction of 3′UTR mutation effect on miRNAs binding.** miRNAs were ranked according to their differential binding to the mutated 3′UTRs by a consensus prediction of the three prediction programs. Top ranked miRNAs are predicted to bind better to the 3′UTR wild type sequences. For the top/bottom 20 miRNAs, a survey of the reported effect in cancer cells is given.
(DOC)

Table S3 **Functional enrichment analysis.** Highlighted biological processes of table S2 top/bottom ranked 20 miRNAs.
(DOC)

Figure S1 **Nucleotide composition plots.** **A.** 3′UTR composition of mutated genes in melanoma, lung cancer and AML. **B.** microRNAs compositions. T represent U.
(DOC)
