# Supplementary material for: Mutagen-Specific Mutation Signature Determines Global microRNA Binding
Source: PLoS One. 2011 Nov 9;6(11):e27400. doi: 10.1371/journal.pone.0027400 (PMC3212558; doi:10.1371/journal.pone.0027400)
Supplement: Table S1 — 3′UTR mutations in a melanoma genome. The genes are sorted by consensus Δb value calculated by the different programs. (DOC) [file pone.0027400.s001.doc]

**Table S1.** 3'UTR mutations in a melanoma genome.

Refseq id symbol mutation details^1^ pita miranda mirhb rank^4^ conser^5^

∆b^2^ rank^3^ ∆b^2^ rank^3^ ∆b^2^ rank^3^ vote -vation

NM_001135254 PAX7 chr1 + 3754 G A 5 14 22 5 226 1 20 0.92

NM_001124758_1 SPNS2 chr17 + 2288 G A 3 31 33 1 145 2 34 0.47

NR_027765 SBF1P1 chr8 - 254 C T 3 28 17 11 95 6 45 0

NM_012455 PSD4 chr2 + 1503 G T 3 25 18 8 56 16 49 0

NM_002235_1 KCNA6 chr12 + 2369 G A 3 29 16 13 72 12 54 0.01

NM_001005473 PLCXD3 chr5 - 5445 C T 2 36 17 10 71 13 59 0

NM_005108 XYLB chr3 + 1219 G A 2 46 13 20 78 10 76 0.22

NM_018263 ASXL2 chr2 - 1779 G T 3 30 11 29 49 26 85 0

NM_018189 DPPA4 chr3 - 783 G T 11 1 5 50 39 35 86 0

NM_001077198 ATG9A chr2 - 921 C T 1 74 19 6 89 8 88 0.99

NM_022114 PRDM16 chr1 + 2840 C A 4 17 4 67 49 25 109 0.00

NM_000966 RARG chr12 - 939 G T 2 43 12 23 19 60 126 0.61

NM_033225 CSMD1 chr8 - 1791 C T 1 56 5 52 52 21 129 0.00

NR_027248_12 ANKRD26P3 chr13 - 38 G A 4 19 11 28 10 84 131 0.87

NM_000782_1 CYP24A1 chr20 - 222 G A 2 50 11 32 23 51 133 0

NM_001105579 TMEM90A chr14 - 222 G A 5 12 12 24 5 107 143 0

NR_003263_6 SDHAP3 chr5 - 273 C T 0 120 11 30 115 3 153 0

NM_005852 CHD3 chr17 + 747 C A 0 123 28 2 43 29 154 1

NR_002791_3 EMX2OS chr10 - 3634 C T 0 100 6 46 89 9 155 0

NM_207416 FLJ44082 chr9 + 82 C T 3 32 4 64 16 63 159 0

NM_178556 TRIML1 chr4 + 93 G A 1 62 4 70 44 28 160 0

NM_001017395 TMCC1 chr3 - 895 G A 5 10 9 38 4 114 162 1

NM_003281_1 TNNI1 chr1 - 175 C T 1 57 4 66 33 42 165 0

NM_024653 PRKRIP1 chr7 + 89 G A 0 135 12 25 64 15 175 0

NM_024627 C22orf29 chr22 - 1220 G A 1 61 5 58 15 66 185 0.00

NM_182705 FAM101B chr17 - 805 G A 1 65 5 57 16 65 187 0

NM_002401 MAP3K3 chr17 + 637 T C 1 63 25 4 1 123 190 0.00

NM_001105204 RUSC1 chr1 + 357 C T 5 6 8 39 -6 146 191 0

NR_027020_4 ANKRD30BL chr2 - 201 G A 0 91 5 53 27 47 191 0

NM_145298 APOBEC3F chr22 + 913 C T 3 27 8 41 1 124 192 0

NM_001136193 FASTKD2 chr2 + 1170 C T 2 35 11 34 1 126 195 0.04

NM_014702 KIAA0408 chr6 - 122 G T 5 7 0 124 16 64 195 1

NM_001012279_5 C6orf174 chr6 - 2528 G T 5 9 0 138 16 62 209 1

NM_032836 FIZ1 chr19 - 885 G A 1 69 5 51 8 90 210 0

NM_012199 EIF2C1 chr1 + 1152 C T 1 73 9 37 7 101 211 0.67

NM_006011 ST8SIA2 chr15 + 2834 C T 6 4 0 136 14 73 213 0

NM_024711 GIMAP6 chr7 - 1435 G A 3 21 10 36 -8 157 214 0

NR_026847_5 LOC286359 chr9 - 852 G A 2 48 12 22 -5 145 215 0.07

NR_024115_2 GIMAP6 chr7 - 2514 G A 3 26 10 35 -8 155 216 0

NM_001130820 RABL5 chr7 - 408 G C 5 8 13 21 -34 189 218 0

NM_001125 ADPRH chr3 + 1540 C T 2 49 3 80 8 89 218 0

NM_006138 MS4A3 chr11 + 386 C T 3 24 1 108 10 87 219 0.00

NM_032204 ASCC2 chr22 - 325 G A 5 11 3 74 -1 136 221 0

NM_003708 RDH16 chr12 - 86 C T -1 169 6 47 96 5 221 0

NM_001085429 TMEM213 chr7 + 929 C A 0 140 4 65 56 18 223 0

NM_001012958 DISC1 chr1 + 147 C T 4 16 5 61 -6 148 225 0

NM_002699 POU3F1 chr1 - 1055 G A 2 38 14 18 -13 169 225 0.83

NM_002837 PTPRB chr12 - 2103 C T 0 139 5 59 43 30 228 0

NM_004275 MED20 chr6 - 654 A T 1 76 0 134 55 19 229 0

NM_002077 GOLGA1 chr9 - 1171 C T 0 141 5 54 39 34 229 0

NM_006413 RPP30 chr10 + 412 C T 1 60 11 27 -3 143 230 0.01

NM_002457 MUC2 chr11 + 54 C T 4 18 3 75 -2 137 230 0.01

NM_005462 MAGEC1 chrX + 364 G A 0 102 1 114 68 14 230 0

NM_181706 DNAJC24 chr11 + 1851 C T 2 42 5 60 0 129 231 0.04

NM_001080395 AATK chr17 - 1012 C A 0 136 3 77 51 23 236 0.00

NM_006627 POP4 chr19 + 909 C T 1 77 8 40 2 120 237 0

NM_014696 GPRIN2 chr10 + 225 C T 1 66 15 15 -8 156 237 0

NM_019044 CCDC93 chr2 - 1886 A T 0 95 1 110 42 32 237 0.00

NM_000899_1 KITLG chr12 - 1547 G A 3 23 0 162 22 54 239 0

NM_000843 GRM6 chr5 - 648 C A -2 192 18 7 37 41 240 0.00

NM_001100166 PHACTR2 chr6 + 6312 C T 1 67 14 17 -9 158 242 0.85

NM_020871 LRCH2 chrX - 1850 G A 2 44 0 128 14 71 243 0.62

NM_133273 FCAR chr19 + 73 G A 0 84 1 111 25 49 244 0.01

NR_024344 LO283174 chr11 - 4548 G A 1 78 13 19 -6 147 244 0

NM_001080433 CCDC85A chr2 + 722 C T 3 22 2 90 0 132 244 0

NR_026714_3 FAM182B chr20 - 2036 G T -1 168 6 48 38 37 253 0

NM_001346 DGKG chr3 - 1205 C T 1 59 0 145 23 53 257 0.01

NM_020152 C21orf7 chr21 + 734 G C 1 54 -12 201 103 4 259 0

NM_004275 MED20 chr6 - 655 A T 2 41 -1 175 31 44 260 0

NM_004447 EPS8 chr12 - 23 G A 0 90 2 95 13 75 260 0

NM_182558 C12orf36 chr12 - 899 G A 0 97 6 45 1 122 264 0.16

NM_207365 AADACL2 chr3 + 58 G A 0 142 1 116 93 7 265 0

NM_024674 LIN28 chr1 + 276 G A -1 162 3 79 50 24 265 0

NM_138361 LRSAM1 chr9 + 429 C T 1 79 26 3 -28 184 266 0

NM_001001931 MTUS1 chr8 - 1241 C T -2 179 4 69 55 20 268 0

NM_021574 BCR chr22 + 1478 G C 3 33 -8 198 38 38 269 0

NM_201279 NRP2 chr2 + 2266 C T -1 152 12 26 8 92 270 0

NM_001142771 PCDH15 chr10 - 1871 G A 2 37 2 93 -2 140 270 0.07

NM_001040078 LGALS9C chr17 + 404 C T 2 34 1 118 2 118 270 0.01

NM_001080391 SP100 chr2 + 2489 G C 2 39 -10 199 41 33 271 0

NM_170735 BDNF chr11 - 1628 C T -3 198 7 43 43 31 272 1

NR_002140 OR6W1P chr7 - 390 G A 1 68 8 42 -14 170 280 0

NM_001145344 ZNF566 chr19 - 2425 C T -1 170 3 85 45 27 282 0.02

NM_133274 FCAR chr19 + 197 G A 0 126 1 106 25 50 282 0.01

NM_032538 TTBK1 chr6 + 1289 C T -1 157 4 73 22 55 285 0

NM_015455 CNOT6 chr5 + 1374 C T 1 52 2 101 0 133 286 0.99

NM_001077263 TMPRSS13 chr11 - 461 G A 2 40 4 68 -23 179 287 0

NM_000899_1 KITLG chr12 - 3860 G A 0 81 0 129 11 79 289 0

NM_001130865_1 DMRT2 chr9 + 805 C T 0 89 1 104 8 96 289 1

NM_000539 RHO chr3 + 1192 C T 0 147 16 14 -1 134 295 0

NM_207333 ZNF320 chr19 - 621 A T 1 72 0 158 15 67 297 0

NM_013243 SCG3 chr15 + 269 G A 0 130 1 121 28 46 297 0.05

NM_005923 MAP3K5 chr6 - 210 C T 0 82 0 161 22 56 299 0.22

NM_001143984 PTPMT1 chr11 + 7 C T 0 94 3 87 2 119 300 0.99

NM_002580 REG3A chr2 - 146 G A 1 80 5 49 -16 171 300 0

NM_004744 LRAT chr4 + 1083 T A 0 111 0 143 27 48 302 0

NM_021628 ALOXE3 chr17 - 584 G A -2 191 14 16 8 98 305 0

NM_020416 PPP2R2C chr4 - 1432 G A 0 144 17 9 -7 154 307 0

NM_014657 KIAA0406 chr20 - 428 C T 1 70 0 132 6 105 307 0.00

NM_024989 PGAP1 chr2 - 5197 C T 0 98 0 159 23 52 309 0

NM_178571_4 TBC1D26 chr17 + 197 G C -3 197 2 98 56 17 312 0

NR_026730_4 TPTE2P1 chr13 - 1191 A T 0 92 -2 186 38 36 314 0.64

NM_006581 FUT9 chr6 + 10343 G A -1 163 0 142 77 11 316 0

NM_003569 STX7 chr6 - 2089 A T 0 83 0 150 10 83 316 0

NM_001144888 BAIAP2 chr17 + 1551 C A -1 166 3 82 15 68 316 0.00

NM_005271 GLUD1 chr10 - 579 G A 6 3 0 147 -10 166 316 1

NM_152381_1 XIRP2 chr2 + 11 C T 0 104 1 103 4 110 317 0.99

NM_017952 PTCD3 chr2 + 330 C T 2 47 2 102 -17 173 322 0

NM_017451 BAIAP2 chr17 + 1085 C A -1 165 3 88 15 70 323 0.00

NM_001033047 NPNT chr4 + 773 C T 0 134 2 96 8 93 323 0.32

NM_002094 GSPT1 chr16 - 3918 C G 0 88 0 164 14 72 324 0.64

NM_001040143 SCN2A chr2 + 2281 C T 0 107 0 141 12 78 326 1

NR_027078_4 LOC283856 chr16 - 590 G A 1 75 4 71 -25 180 326 0

NM_002994 CXCL5 chr4 - 439 C T 0 117 0 154 21 57 328 0

NM_001040078 LGALS9C chr17 + 411 C A -4 202 3 83 32 43 328 0.00

NM_006557 DMRT2 chr9 + 1221 C T 0 133 1 105 8 91 329 1

NM_006340_1 BAIAP2 chr17 + 1639 C A -1 171 3 89 15 69 329 0.00

NM_025010 KLHL18 chr3 + 278 C T 0 113 5 55 -9 161 329 0.00

NM_001010924 FAM171A1 chr10 - 783 C A 0 112 1 117 7 100 329 0

NM_001044370 MPPED1 chr22 + 31 C A 0 119 -4 191 52 22 332 0

NM_207330 NPAL1 chr4 + 410 C T 0 118 0 126 9 88 332 0.00

NM_024744 ALS2CR8 chr2 + 599 C T 7 2 -2 180 -7 150 332 0

NM_001136103 TMEM132C chr12 + 1516 G A 0 125 0 149 20 58 332 0.00

NR_022006_1 KIAA0087 chr7 - 1969 C T 0 150 0 137 29 45 332 0

NM_001742 CALCR chr7 - 1223 C T 0 129 0 148 19 61 338 0.65

NM_000508 FGA chr4 - 333 G A 0 127 2 94 2 117 338 0

NM_002580 REG3A chr2 - 130 G C 2 45 2 99 -46 196 340 0

NM_001003714 ATP5J2 chr7 - 110 G A 0 116 1 120 6 104 340 0

NM_006948 HSPA13 chr21 - 1907 G A -1 160 3 78 7 103 341 0

NM_001099771 POTEF chr2 - 8 G A 0 106 0 140 8 95 341 0

NM_003144 SSR1 chr6 - 3399 G A 1 58 2 100 -36 190 348 0

NR_024430_3 MIR100HG chr11 - 727 G A 5 13 -1 168 -11 167 348 0.49

NM_001162501 TNRC6B chr22 + 5791 C A 0 87 0 135 0 127 349 0.05

NM_023943 TMEM108 chr3 + 1141 T C -3 196 5 56 7 99 351 0

NM_001040653 ZXDC chr3 - 429 A T 0 114 0 123 3 116 353 0.00

NM_006334 OLFM1 chr9 + 234 G A 0 110 0 146 8 97 353 0.75

NM_001080395 AATK chr17 - 330 A G -4 203 3 76 13 74 353 0

NM_170665 ATP2A2 chr12 + 2111 C T -1 159 11 33 -9 162 354 0.00

NM_001126117_2 TP53 chr17 - 1360 G A 0 99 -1 172 10 85 356 0.22

NM_001547 IFIT2 chr10 + 1220 C T 0 105 -1 177 12 77 359 0

NM_001126116_2 TP53 chr17 - 1448 G A 0 103 -1 176 10 82 361 0.22

NR_027120_2 HSD52 chr1 - 686 G A 1 71 0 152 -2 139 362 0

NM_001059 TACR3 chr4 - 9 G A 0 115 1 112 -2 138 365 0

NM_130474_1 MADD chr11 + 43 C T -6 206 3 86 12 76 368 0.99

NM_023078 PYCRL chr8 - 1108 C G -2 182 7 44 -3 142 368 0

NM_004293 GDA chr9 + 3233 G A -1 164 0 165 38 39 368 0

NM_000912 OPRK1 chr8 - 1470 G A -2 177 1 113 11 80 370 0

NM_001135765 ATP2A2 chr12 + 2111 C T -1 176 11 31 -9 163 370 0.00

NR_003129 RNF5P1 chr8 - 1057 C T 0 124 0 131 3 115 370 0.14

NM_130807 MOBKL2A chr19 - 2111 G A -2 186 5 63 2 121 370 0

NR_024430_3 MIR100HG chr11 - 2977 A G 0 101 -2 188 10 81 370 0.02

NM_001419 ELAVL1 chr19 - 3932 A T -1 156 0 156 20 59 371 0.04

NM_014553 TFCP2L1 chr2 - 6951 C A -4 201 5 62 4 112 375 0.00

NM_033661_1 WDR4 chr21 - 32 G A -6 205 16 12 -9 159 376 0

NM_139067 SMARCC2 chr12 - 84 G A 5 5 -6 196 -22 177 378 0.99

NM_006358 SLC25A17 chr22 - 355 G A -1 158 2 92 0 128 378 0

NM_144582 TEX261 chr2 - 1062 G A -1 175 4 72 -1 135 382 0.99

NM_003189 TAL1 chr1 - 907 G C 4 15 -6 195 -21 174 384 0

NM_001126115 TP53 chr17 - 1159 G A 0 128 -1 171 10 86 385 0.22

NM_013255 MKLN1 chr7 + 4531 C T -2 184 2 97 5 108 389 0

NM_005666 CFHR2 chr1 + 71 C T 0 137 0 166 8 94 397 0

NR_024380_2 LOC441666 chr10 - 2936 G C 0 109 1 115 -21 175 399 0.00

NM_001142351 ST6GAL2 chr2 - 2419 T C 4 20 -4 192 -37 191 403 0.01

NM_007137 ZNF81 chrX + 1539 C T 0 132 2 91 -25 181 404 0

NM_001024843 TNRC6B chr22 + 5791 C A 0 122 0 153 0 131 406 0.05

NM_004227 CYTH3 chr7 - 5 G A 0 149 0 125 -2 141 415 0.19

NM_144644 SPATA4 chr4 - 110 G A 0 121 1 109 -38 192 422 0.07

NM_173598 KSR2 chr12 - 1820 G T -4 200 -2 185 37 40 425 0.00

NR_027995 LO284232 chr13 - 28980 T C 0 96 0 130 -54 200 426 0.00

NM_145259 ACVR1C chr2 - 778 G A -6 204 3 84 -3 144 432 0

NM_022167 XYLT2 chr17 + 260 A T 0 138 1 107 -31 188 433 0.00

NM_005056_1 KDM5A chr12 - 5081 T A 1 51 -2 187 -45 195 433 0

NM_001040431 CCDC56 chr17 - 117 G A -2 185 3 81 -16 172 438 0

NM_001042603 JARID1A chr12 - 4837 T A 1 64 -2 184 -45 194 442 0

NM_001014283 DCUN1D2 chr13 - 147 T G 1 55 -12 202 -29 185 442 0

NM_003189 TAL1 chr1 - 908 G A -1 167 -1 170 5 106 443 0

NR_027995 LO284232 chr13 - 28813 G A -2 181 0 160 7 102 443 0.05

NM_033055 HIAT1 chr1 + 1108 A T 0 146 0 151 -7 153 450 0.99

NM_007123 USH2A chr1 - 381 G A -1 161 -2 183 5 109 453 0

NM_024969 CSRNP3 chr2 + 260 T C -1 151 -4 193 4 111 455 1

NM_004257 TGFBRAP1 chr2 - 298 C T -2 183 0 155 1 125 463 0

NM_182775 ALS2CL chr3 - 884 T C 1 53 -15 204 -134 208 465 0.00

NM_001037293 PALM2 chr9 + 7575 C T -1 174 -2 181 4 113 468 0.16

NM_001040101 D4S234E chr4 + 201 T C 0 131 -3 189 -7 149 469 0.63

NM_001142413 C9ORF47 chr9 + 1605 A G 0 108 -1 174 -83 204 486 0

NM_014392 D4S234E chr4 + 201 T C 0 148 -3 190 -7 152 490 0.63

NM_017812 CHCHD3 chr7 - 726 A C -1 154 -1 173 -10 164 491 0.48

NM_001003652 SMAD2 chr18 - 271 G A -3 195 1 119 -22 178 492 1

NM_001135937 SMAD2 chr18 - 271 G A -3 194 1 122 -22 176 492 1

NM_004613 TGM2 chr20 - 1389 G A 0 143 -12 200 -7 151 494 0.00

NM_001029871 RSPO4 chr20 - 712 A C 0 85 -30 207 -103 205 497 0

NM_001042476 CARHSP1 chr16 - 804 A C 0 86 -17 205 -131 207 498 0.00

NM_015566 FAM169A chr5 - 3159 T G -1 155 0 163 -28 183 501 0.96

NM_020792 AADACL1 chr3 - 866 G A -11 208 0 127 -13 168 503 0.14

NM_017679 BCAS3 chr17 + 46 A G 0 93 -32 208 -117 206 507 0.16

NM_005870 SAP18 chr13 + 37 A G 0 145 -1 167 -48 197 509 0.96

NM_001975 ENO2 chr12 + 495 T G -2 190 0 139 -27 182 511 0

NM_007138 ZNF90 chr19 + 1201 T C -2 187 -1 169 -9 160 516 0

NM_018048 MAGOHB chr12 - 1819 G A -2 180 -1 178 -10 165 523 0

NM_015292 FAM62A chr12 + 435 T C -6 207 -5 194 0 130 531 0

NM_001135733_1 TP53INP1 chr8 - 3100 T C -2 189 0 144 -54 199 532 0.00

NM_033285 TP53INP1 chr8 - 2834 T C -2 178 0 157 -54 198 533 0.00

NM_014506 TOR1B chr9 + 775 T G -3 199 0 133 -78 202 534 0.06

NM_173082 SHPRH chr6 - 1361 A C -1 172 -2 179 -30 186 537 0

NM_001042683 SHPRH chr6 - 1624 A C -1 173 -2 182 -30 187 542 0

NM_152879 DGKD chr2 + 250 A C -1 153 -14 203 -40 193 549 0

NM_021165 FAM5B chr1 + 759 A G -2 188 -7 197 -64 201 586 0.98

NM_017588 WDR5 chr9 + 78 T G -3 193 -28 206 -82 203 602 0

1. Mutation details include: chr, strand, location in the gene, wild type base, mutation base all separated by spaces.

2. ∆b = number of miRNAs bind to wild type sequence minus number of miRNAs bind to mutant sequence.

3. The rank position after sorting the ∆b values in decreasing order.

4. A weighted consensus rank of the 3 programs.

5. Conservation score of the mutation position.

**Table S1.** 3'UTR mutations in a melanoma genome. The genes are sorted by consensus ∆b value calculated by the different programs.
