# Supplementary material for: Mutagen-Specific Mutation Signature Determines Global microRNA Binding
Source: PLoS One. 2011 Nov 9;6(11):e27400. doi: 10.1371/journal.pone.0027400 (PMC3212558; doi:10.1371/journal.pone.0027400)
Supplement: Table S2 — Prediction of 3′UTR mutation effect on miRNAs binding. miRNAs were ranked according to their differential binding to the mutated 3′UTRs by a consensus prediction of the three prediction programs. Top ranked miRNAs are predicted to bind better to the 3′UTR wild type sequences. For the top/bottom 20 miRNAs, a survey of the reported effect in cancer cells is given. (DOC) [file pone.0027400.s002.doc]

**Table S2. Prediction of 3'UTR mutation effect on miRNAs binding.**

| microRNA | ∆b pita | rank pita | ∆b miranda | rank miranda | ∆b mirhb | rank mirhb | normalized consensus rank | Reported effect | |
| --- | --- | --- | --- | --- | --- | --- | --- | --- | --- |
|  |  |  |  |  |  |  |  | Oncogenic/ upregulated | Suppressor/ downregulated |
| hsa-miR-658 | 8 | 3 | 12 | 4 | 40 | 4 | 0.317935 |  |  |
| hsa-miR-1909 | 9 | 2 | 9 | 7 | 35 | 6 | 0.427536 |  |  |
| hsa-miR-1207-5p | 3 | 8 | 15 | 3 | 46 | 3 | 0.566123 |  |  |
| hsa-miR-663 | 7 | 4 | 8 | 8 | 35 | 6 | 0.596014 |  | , |
| hsa-miR-762 | 4 | 7 | 10 | 6 | 52 | 2 | 0.613225 |  |  |
| hsa-miR-939 | 2 | 9 | 20 | 1 | 34 | 7 | 0.625 |  |  |
| hsa-miR-638 | 4 | 7 | 11 | 5 | 31 | 9 | 0.71558 |  |  |
| hsa-miR-608 | -1 | 12 | 12 | 4 | 60 | 1 | 0.817935 |  |  |
| hsa-miR-615-5p | 3 | 8 | 17 | 2 | 15 | 20 | 0.876812 |  |  |
| hsa-miR-1268 | 1 | 10 | 12 | 4 | 26 | 12 | 0.922101 |  |  |
| hsa-miR-940 | 6 | 5 | 5 | 11 | 20 | 16 | 0.997283 |  |  |
| hsa-miR-432 | 0 | 11 | 11 | 5 | 27 | 11 | 1.007246 |  |  |
| hsa-miR-1908 | 2 | 9 | 6 | 10 | 32 | 8 | 1.037138 |  |  |
| hsa-miR-184 | 8 | 3 | 3 | 13 | 14 | 21 | 1.063406 |  | ,, |
| hsa-miR-886-5p | 3 | 8 | 1 | 15 | 40 | 4 | 1.108696 |  |  |
| hsa-miR-491-5p | 6 | 5 | 6 | 10 | 8 | 27 | 1.182971 |  |  |
| hsa-miR-659 | 1 | 10 | 12 | 4 | 10 | 25 | 1.192935 |  |  |
| hsa-miR-1469 | 0 | 11 | 8 | 8 | 24 | 14 | 1.200181 |  |  |
| hsa-miR-765 | 5 | 6 | 2 | 14 | 19 | 17 | 1.211051 |  |  |
| hsa-miR-1538 | 3 | 8 | -1 | 17 | 36 | 5 | 1.216486 |  |  |
| hsa-miR-483-5p | 3 | 8 | 8 | 8 | 11 | 24 | 1.221014 |  |  |
| hsa-miR-625 | 11 | 1 | 0 | 16 | 6 | 29 | 1.235507 |  |  |
| hsa-miR-650 | 7 | 4 | 7 | 9 | 0 | 35 | 1.243659 |  |  |
| hsa-miR-665 | 4 | 7 | 8 | 8 | 5 | 30 | 1.283514 |  |  |
| hsa-miR-1293 | 1 | 10 | 6 | 10 | 17 | 18 | 1.307971 |  |  |
| hsa-miR-612 | -2 | 13 | 5 | 11 | 34 | 7 | 1.309783 |  |  |
| hsa-miR-661 | 0 | 11 | 1 | 15 | 36 | 5 | 1.317029 |  |  |
| hsa-miR-1182 | -1 | 12 | 7 | 9 | 22 | 15 | 1.326993 |  |  |
| hsa-miR-324-3p | 2 | 9 | 7 | 9 | 11 | 24 | 1.326993 |  |  |
| hsa-miR-663b | 2 | 9 | 7 | 9 | 11 | 24 | 1.326993 |  |  |
| hsa-miR-623 | 1 | 10 | 9 | 7 | 9 | 26 | 1.344203 |  |  |
| hsa-miR-637 | -2 | 13 | 7 | 9 | 25 | 13 | 1.347826 |  |  |
| hsa-miR-1292 | 3 | 8 | 7 | 9 | 7 | 28 | 1.347826 |  |  |
| hsa-miR-601 | 2 | 9 | 3 | 13 | 17 | 18 | 1.375906 |  |  |
| hsa-miR-760 | 0 | 11 | 9 | 7 | 10 | 25 | 1.38587 |  |  |
| hsa-miR-1247 | 4 | 7 | 3 | 13 | 10 | 25 | 1.396739 |  |  |
| hsa-miR-330-5p | 2 | 9 | 1 | 15 | 22 | 15 | 1.400362 |  |  |
| hsa-miR-744 | 2 | 9 | 4 | 12 | 13 | 22 | 1.415761 |  |  |
| hsa-miR-1266 | 1 | 10 | 2 | 14 | 22 | 15 | 1.419384 |  |  |
| hsa-miR-1248 | 1 | 10 | 1 | 15 | 24 | 14 | 1.442029 |  |  |
| hsa-miR-541 | 2 | 9 | 1 | 15 | 19 | 17 | 1.442029 |  |  |
| hsa-miR-1471 | 1 | 10 | 7 | 9 | 8 | 27 | 1.451993 |  |  |
| hsa-miR-370 | 1 | 10 | 11 | 5 | -1 | 36 | 1.46558 |  |  |
| hsa-miR-1915 | -1 | 12 | 9 | 7 | 9 | 26 | 1.469203 |  |  |
| hsa-miR-453 | 1 | 10 | 4 | 12 | 13 | 22 | 1.478261 |  |  |
| hsa-miR-296-3p | 0 | 11 | 10 | 6 | 3 | 32 | 1.488225 |  |  |
| hsa-miR-423-3p | 0 | 11 | 6 | 10 | 11 | 24 | 1.495471 |  |  |
| hsa-miR-193a-5p | -2 | 13 | 2 | 14 | 28 | 10 | 1.502717 |  |  |
| hsa-miR-1913 | 1 | 10 | 2 | 14 | 16 | 19 | 1.502717 |  |  |
| hsa-miR-138 | 1 | 10 | 1 | 15 | 19 | 17 | 1.504529 |  |  |
| hsa-miR-675 | 1 | 10 | 0 | 16 | 22 | 15 | 1.506341 |  |  |
| hsa-miR-339-5p | 2 | 9 | 9 | 7 | -2 | 37 | 1.51087 |  |  |
| hsa-miR-769-3p | 0 | 11 | 8 | 8 | 6 | 29 | 1.512681 |  |  |
| hsa-miR-134 | 1 | 10 | 7 | 9 | 5 | 30 | 1.514493 |  |  |
| hsa-miR-2110 | 3 | 8 | 2 | 14 | 9 | 26 | 1.523551 |  |  |
| hsa-miR-423-5p | 3 | 8 | -1 | 17 | 15 | 20 | 1.528986 |  |  |
| hsa-miR-1225-5p | -2 | 13 | 10 | 6 | 7 | 28 | 1.529891 |  |  |
| hsa-miR-331-5p | 1 | 10 | 3 | 13 | 12 | 23 | 1.542572 |  |  |
| hsa-miR-1202 | 0 | 11 | 9 | 7 | 2 | 33 | 1.552536 |  |  |
| hsa-miR-636 | 0 | 11 | 5 | 11 | 10 | 25 | 1.559783 |  |  |
| hsa-miR-770-5p | 2 | 9 | 4 | 12 | 6 | 29 | 1.561594 |  |  |
| hsa-miR-185 | 0 | 11 | 3 | 13 | 14 | 21 | 1.563406 |  |  |
| hsa-miR-1321 | 5 | 6 | 1 | 15 | 3 | 32 | 1.567029 |  |  |
| hsa-miR-323-5p | 5 | 6 | -4 | 20 | 13 | 22 | 1.576087 |  |  |
| hsa-miR-1180 | -1 | 12 | 4 | 12 | 14 | 21 | 1.582428 |  |  |
| hsa-miR-1291 | -1 | 12 | 4 | 12 | 14 | 21 | 1.582428 |  |  |
| hsa-miR-933 | -1 | 12 | 4 | 12 | 14 | 21 | 1.582428 |  |  |
| hsa-miR-1203 | 2 | 9 | 3 | 13 | 7 | 28 | 1.584239 |  |  |
| hsa-miR-642 | 1 | 10 | 2 | 14 | 12 | 23 | 1.586051 |  |  |
| hsa-miR-1231 | 1 | 10 | 2 | 14 | 12 | 23 | 1.586051 |  |  |
| hsa-miR-1910 | 2 | 9 | 2 | 14 | 9 | 26 | 1.586051 |  |  |
| hsa-miR-513a-5p | 4 | 7 | 1 | 15 | 5 | 30 | 1.587862 |  |  |
| hsa-miR-1255b | 0 | 11 | 1 | 15 | 17 | 18 | 1.587862 |  |  |
| hsa-miR-1249 | 4 | 7 | 0 | 16 | 7 | 28 | 1.589674 |  |  |
| hsa-miR-557 | 0 | 11 | 3 | 13 | 12 | 23 | 1.605072 |  |  |
| hsa-miR-887 | 1 | 10 | 3 | 13 | 9 | 26 | 1.605072 |  |  |
| hsa-miR-542-5p | 0 | 11 | 2 | 14 | 14 | 21 | 1.606884 |  |  |
| hsa-miR-449b | 0 | 11 | 1 | 15 | 16 | 19 | 1.608696 |  |  |
| hsa-miR-1976 | 3 | 8 | 0 | 16 | 9 | 26 | 1.610507 |  |  |
| hsa-miR-611 | -1 | 12 | 9 | 7 | 2 | 33 | 1.615036 |  |  |
| hsa-miR-602 | 1 | 10 | 5 | 11 | 4 | 31 | 1.622283 |  |  |
| hsa-miR-718 | 2 | 9 | 3 | 13 | 5 | 30 | 1.625906 |  |  |
| hsa-miR-1269 | 0 | 11 | 2 | 14 | 13 | 22 | 1.627717 |  |  |
| hsa-miR-1225-3p | 0 | 11 | 2 | 14 | 13 | 22 | 1.627717 |  |  |
| hsa-miR-211 | 0 | 11 | 1 | 15 | 15 | 20 | 1.629529 |  |  |
| hsa-miR-1262 | 0 | 11 | 0 | 16 | 17 | 18 | 1.631341 |  |  |
| hsa-miR-486-3p | 2 | 9 | -2 | 18 | 15 | 20 | 1.634964 |  |  |
| hsa-miR-331-3p | 0 | 11 | 6 | 10 | 4 | 31 | 1.641304 |  |  |
| hsa-miR-346 | 0 | 11 | 5 | 11 | 6 | 29 | 1.643116 |  |  |
| hsa-miR-1287 | 1 | 10 | 3 | 13 | 7 | 28 | 1.646739 |  |  |
| hsa-miR-345 | 0 | 11 | 2 | 14 | 12 | 23 | 1.648551 |  |  |
| hsa-miR-874 | 1 | 10 | 1 | 15 | 11 | 24 | 1.650362 |  |  |
| hsa-miR-508-3p | 0 | 11 | 1 | 15 | 14 | 21 | 1.650362 |  |  |
| hsa-miR-671-3p | 0 | 11 | 3 | 13 | 9 | 26 | 1.667572 |  |  |
| hsa-miR-361-3p | 1 | 10 | 2 | 14 | 8 | 27 | 1.669384 |  |  |
| hsa-miR-18b | 2 | 9 | 1 | 15 | 7 | 28 | 1.671196 |  |  |
| hsa-miR-1914 | 0 | 11 | 0 | 16 | 15 | 20 | 1.673007 |  |  |
| hsa-miR-574-5p | 1 | 10 | -1 | 17 | 14 | 21 | 1.674819 |  |  |
| hsa-miR-154 | 0 | 11 | -1 | 17 | 17 | 18 | 1.674819 |  |  |
| hsa-miR-662 | 0 | 11 | 6 | 10 | 2 | 33 | 1.682971 |  |  |
| hsa-miR-1301 | 0 | 11 | 5 | 11 | 4 | 31 | 1.684783 |  |  |
| hsa-miR-657 | -2 | 13 | 5 | 11 | 10 | 25 | 1.684783 |  |  |
| hsa-miR-490-5p | 0 | 11 | 4 | 12 | 6 | 29 | 1.686594 |  |  |
| hsa-miR-615-3p | -1 | 12 | 4 | 12 | 9 | 26 | 1.686594 |  |  |
| hsa-miR-520a-5p | 1 | 10 | 3 | 13 | 5 | 30 | 1.688406 |  |  |
| hsa-miR-188-5p | 0 | 11 | 3 | 13 | 8 | 27 | 1.688406 |  |  |
| hsa-miR-1275 | 2 | 9 | 2 | 14 | 4 | 31 | 1.690217 |  |  |
| hsa-miR-206 | -1 | 12 | 1 | 15 | 15 | 20 | 1.692029 |  |  |
| hsa-miR-1238 | 1 | 10 | 1 | 15 | 9 | 26 | 1.692029 |  |  |
| hsa-miR-1255a | 0 | 11 | 0 | 16 | 14 | 21 | 1.693841 |  |  |
| hsa-miR-328 | 1 | 10 | 0 | 16 | 11 | 24 | 1.693841 |  |  |
| hsa-miR-338-3p | 0 | 11 | 0 | 16 | 14 | 21 | 1.693841 |  |  |
| hsa-miR-1224-5p | -1 | 12 | 8 | 8 | 0 | 35 | 1.700181 |  |  |
| hsa-miR-645 | 1 | 10 | 3 | 13 | 4 | 31 | 1.709239 |  |  |
| hsa-miR-375 | 0 | 11 | 3 | 13 | 7 | 28 | 1.709239 |  |  |
| hsa-miR-1308 | 0 | 11 | 3 | 13 | 7 | 28 | 1.709239 |  |  |
| hsa-miR-2114 | 1 | 10 | 3 | 13 | 4 | 31 | 1.709239 |  |  |
| hsa-miR-1307 | -1 | 12 | 2 | 14 | 12 | 23 | 1.711051 |  |  |
| hsa-miR-941 | 1 | 10 | 2 | 14 | 6 | 29 | 1.711051 |  |  |
| hsa-miR-605 | 1 | 10 | 1 | 15 | 8 | 27 | 1.712862 |  |  |
| hsa-miR-595 | 2 | 9 | 1 | 15 | 5 | 30 | 1.712862 |  |  |
| hsa-miR-526b | -1 | 12 | 1 | 15 | 14 | 21 | 1.712862 |  |  |
| hsa-miR-145 | 0 | 11 | 0 | 16 | 13 | 22 | 1.714674 |  |  |
| hsa-miR-1468 | 0 | 11 | -1 | 17 | 15 | 20 | 1.716486 |  |  |
| hsa-miR-942 | 1 | 10 | -2 | 18 | 14 | 21 | 1.718297 |  |  |
| hsa-miR-188-3p | 0 | 11 | 6 | 10 | 0 | 35 | 1.724638 |  |  |
| hsa-miR-210 | 0 | 11 | 4 | 12 | 4 | 31 | 1.728261 |  |  |
| hsa-miR-566 | 0 | 11 | 4 | 12 | 4 | 31 | 1.728261 |  |  |
| hsa-miR-1228 | 1 | 10 | 4 | 12 | 1 | 34 | 1.728261 |  |  |
| hsa-miR-711 | 0 | 11 | 3 | 13 | 6 | 29 | 1.730072 |  |  |
| hsa-miR-571 | 1 | 10 | 1 | 15 | 7 | 28 | 1.733696 |  |  |
| hsa-miR-18a | 2 | 9 | 1 | 15 | 4 | 31 | 1.733696 |  |  |
| hsa-miR-433 | -1 | 12 | 1 | 15 | 13 | 22 | 1.733696 |  |  |
| hsa-miR-1296 | 1 | 10 | -1 | 17 | 11 | 24 | 1.737319 |  |  |
| hsa-miR-508-5p | -1 | 12 | 5 | 11 | 4 | 31 | 1.747283 |  |  |
| hsa-miR-28-3p | 1 | 10 | 2 | 14 | 4 | 31 | 1.752717 |  |  |
| hsa-miR-501-5p | 1 | 10 | 2 | 14 | 4 | 31 | 1.752717 |  |  |
| hsa-miR-639 | 0 | 11 | 2 | 14 | 7 | 28 | 1.752717 |  |  |
| hsa-miR-1250 | 0 | 11 | 2 | 14 | 7 | 28 | 1.752717 |  |  |
| hsa-miR-518d-5p | 0 | 11 | 1 | 15 | 9 | 26 | 1.754529 |  |  |
| hsa-miR-450b-3p | 2 | 9 | 1 | 15 | 3 | 32 | 1.754529 |  |  |
| hsa-miR-520c-5p | 0 | 11 | 1 | 15 | 9 | 26 | 1.754529 |  |  |
| hsa-miR-526a | 0 | 11 | 1 | 15 | 9 | 26 | 1.754529 |  |  |
| hsa-miR-509-3p | 3 | 8 | 0 | 16 | 2 | 33 | 1.756341 |  |  |
| hsa-miR-1237 | 0 | 11 | 5 | 11 | 0 | 35 | 1.768116 |  |  |
| hsa-miR-193b | 0 | 11 | 4 | 12 | 2 | 33 | 1.769928 |  |  |
| hsa-miR-513c | -1 | 12 | 3 | 13 | 7 | 28 | 1.771739 |  |  |
| hsa-miR-1233 | 0 | 11 | 3 | 13 | 4 | 31 | 1.771739 |  |  |
| hsa-miR-484 | 0 | 11 | 2 | 14 | 6 | 29 | 1.773551 |  |  |
| hsa-miR-504 | 0 | 11 | 2 | 14 | 6 | 29 | 1.773551 |  |  |
| hsa-miR-490-3p | 0 | 11 | 1 | 15 | 8 | 27 | 1.775362 |  |  |
| hsa-miR-593 | 0 | 11 | 0 | 16 | 10 | 25 | 1.777174 |  |  |
| hsa-miR-422a | 2 | 9 | 0 | 16 | 4 | 31 | 1.777174 |  |  |
| hsa-miR-1972 | 1 | 10 | 0 | 16 | 7 | 28 | 1.777174 |  |  |
| hsa-miR-1470 | 1 | 10 | -1 | 17 | 9 | 26 | 1.778986 |  |  |
| hsa-miR-892b | -1 | 12 | 3 | 13 | 6 | 29 | 1.792572 |  |  |
| hsa-miR-329 | 1 | 10 | 3 | 13 | 0 | 35 | 1.792572 |  |  |
| hsa-miR-604 | 3 | 8 | 2 | 14 | -4 | 39 | 1.794384 |  |  |
| hsa-miR-489 | 0 | 11 | 2 | 14 | 5 | 30 | 1.794384 |  |  |
| hsa-miR-151-3p | 2 | 9 | 1 | 15 | 1 | 34 | 1.796196 |  |  |
| hsa-miR-1299 | 0 | 11 | 1 | 15 | 7 | 28 | 1.796196 |  |  |
| hsa-miR-204 | 0 | 11 | 0 | 16 | 9 | 26 | 1.798007 |  |  |
| hsa-miR-455-5p | 0 | 11 | 0 | 16 | 9 | 26 | 1.798007 |  |  |
| hsa-miR-149 | 0 | 11 | 0 | 16 | 9 | 26 | 1.798007 |  |  |
| hsa-miR-635 | 2 | 9 | -1 | 17 | 5 | 30 | 1.799819 |  |  |
| hsa-miR-147b | 0 | 11 | -1 | 17 | 11 | 24 | 1.799819 |  |  |
| hsa-miR-205 | -1 | 12 | 6 | 10 | -1 | 36 | 1.807971 |  |  |
| hsa-miR-564 | 0 | 11 | 5 | 11 | -2 | 37 | 1.809783 |  |  |
| hsa-miR-500 | 0 | 11 | 3 | 13 | 2 | 33 | 1.813406 |  |  |
| hsa-miR-1207-3p | 0 | 11 | 2 | 14 | 4 | 31 | 1.815217 |  |  |
| hsa-miR-1323 | 0 | 11 | 2 | 14 | 4 | 31 | 1.815217 |  |  |
| hsa-miR-1295 | 0 | 11 | 2 | 14 | 4 | 31 | 1.815217 |  |  |
| hsa-miR-196b | -1 | 12 | 2 | 14 | 7 | 28 | 1.815217 |  |  |
| hsa-miR-524-5p | 3 | 8 | 1 | 15 | -3 | 38 | 1.817029 |  |  |
| hsa-miR-125b | -1 | 12 | 1 | 15 | 9 | 26 | 1.817029 |  |  |
| hsa-miR-23b | 0 | 11 | 1 | 15 | 6 | 29 | 1.817029 |  |  |
| hsa-miR-1263 | 1 | 10 | 1 | 15 | 3 | 32 | 1.817029 |  |  |
| hsa-miR-342-5p | -1 | 12 | 1 | 15 | 9 | 26 | 1.817029 |  |  |
| hsa-miR-505 | 0 | 11 | 1 | 15 | 6 | 29 | 1.817029 |  |  |
| hsa-miR-1974 | 1 | 10 | 0 | 16 | 5 | 30 | 1.818841 |  |  |
| hsa-miR-511 | 0 | 11 | 0 | 16 | 8 | 27 | 1.818841 |  |  |
| hsa-miR-409-5p | 0 | 11 | 0 | 16 | 8 | 27 | 1.818841 |  |  |
| hsa-miR-365 | 0 | 11 | 0 | 16 | 8 | 27 | 1.818841 |  |  |
| hsa-miR-520d-5p | 3 | 8 | 0 | 16 | -1 | 36 | 1.818841 |  |  |
| hsa-miR-326 | 1 | 10 | -1 | 17 | 7 | 28 | 1.820652 |  |  |
| hsa-miR-548q | 2 | 9 | -2 | 18 | 6 | 29 | 1.822464 |  |  |
| hsa-miR-670 | 1 | 10 | -2 | 18 | 9 | 26 | 1.822464 |  |  |
| hsa-miR-885-5p | 0 | 11 | 5 | 11 | -3 | 38 | 1.830616 |  |  |
| hsa-miR-193a-3p | 0 | 11 | 4 | 12 | -1 | 36 | 1.832428 |  |  |
| hsa-miR-325 | 0 | 11 | 3 | 13 | 1 | 34 | 1.834239 |  |  |
| hsa-miR-501-3p | 0 | 11 | 3 | 13 | 1 | 34 | 1.834239 |  |  |
| hsa-miR-1289 | 1 | 10 | 2 | 14 | 0 | 35 | 1.836051 |  |  |
| hsa-miR-217 | 1 | 10 | 2 | 14 | 0 | 35 | 1.836051 |  |  |
| hsa-miR-619 | 0 | 11 | 2 | 14 | 3 | 32 | 1.836051 |  |  |
| hsa-miR-92a | 0 | 11 | 2 | 14 | 3 | 32 | 1.836051 |  |  |
| hsa-miR-9 | 0 | 11 | 1 | 15 | 5 | 30 | 1.837862 |  |  |
| hsa-miR-1288 | 0 | 11 | 1 | 15 | 5 | 30 | 1.837862 |  |  |
| hsa-miR-199a-5p | 0 | 11 | 1 | 15 | 5 | 30 | 1.837862 |  |  |
| hsa-miR-621 | 0 | 11 | 1 | 15 | 5 | 30 | 1.837862 |  |  |
| hsa-miR-140-5p | 1 | 10 | 0 | 16 | 4 | 31 | 1.839674 |  |  |
| hsa-miR-200b | 1 | 10 | 0 | 16 | 4 | 31 | 1.839674 |  |  |
| hsa-miR-220a | 1 | 10 | 0 | 16 | 4 | 31 | 1.839674 |  |  |
| hsa-let-7e | 0 | 11 | 0 | 16 | 7 | 28 | 1.839674 |  |  |
| hsa-miR-580 | 1 | 10 | 0 | 16 | 4 | 31 | 1.839674 |  |  |
| hsa-miR-197 | 2 | 9 | 0 | 16 | 1 | 34 | 1.839674 |  |  |
| hsa-miR-122 | 1 | 10 | 0 | 16 | 4 | 31 | 1.839674 |  |  |
| hsa-miR-1282 | 0 | 11 | -1 | 17 | 9 | 26 | 1.841486 |  |  |
| hsa-miR-631 | 1 | 10 | -2 | 18 | 8 | 27 | 1.843297 |  |  |
| hsa-miR-130a | 0 | 11 | 3 | 13 | 0 | 35 | 1.855072 |  |  |
| hsa-miR-324-5p | 0 | 11 | 3 | 13 | 0 | 35 | 1.855072 |  |  |
| hsa-miR-212 | 0 | 11 | 2 | 14 | 2 | 33 | 1.856884 |  |  |
| hsa-miR-150 | 0 | 11 | 2 | 14 | 2 | 33 | 1.856884 |  |  |
| hsa-miR-20b | 0 | 11 | 2 | 14 | 2 | 33 | 1.856884 |  |  |
| hsa-miR-1254 | -1 | 12 | 1 | 15 | 7 | 28 | 1.858696 |  |  |
| hsa-miR-221 | -1 | 12 | 1 | 15 | 7 | 28 | 1.858696 |  |  |
| hsa-miR-935 | 0 | 11 | 1 | 15 | 4 | 31 | 1.858696 |  |  |
| hsa-miR-1257 | 0 | 11 | 1 | 15 | 4 | 31 | 1.858696 |  |  |
| hsa-let-7b | -2 | 13 | 1 | 15 | 10 | 25 | 1.858696 |  |  |
| hsa-miR-1273 | 0 | 11 | 1 | 15 | 4 | 31 | 1.858696 |  |  |
| hsa-miR-425 | 0 | 11 | 1 | 15 | 4 | 31 | 1.858696 |  |  |
| hsa-miR-647 | 0 | 11 | 0 | 16 | 6 | 29 | 1.860507 |  |  |
| hsa-miR-1825 | 0 | 11 | 0 | 16 | 6 | 29 | 1.860507 |  |  |
| hsa-miR-937 | 0 | 11 | 0 | 16 | 6 | 29 | 1.860507 |  |  |
| hsa-miR-502-5p | 1 | 10 | 0 | 16 | 3 | 32 | 1.860507 |  |  |
| hsa-miR-146b-5p | 2 | 9 | 0 | 16 | 0 | 35 | 1.860507 |  |  |
| hsa-miR-451 | 0 | 11 | 0 | 16 | 6 | 29 | 1.860507 |  |  |
| hsa-miR-1245 | 1 | 10 | 0 | 16 | 3 | 32 | 1.860507 |  |  |
| hsa-miR-486-5p | 0 | 11 | 0 | 16 | 6 | 29 | 1.860507 |  |  |
| hsa-miR-632 | -1 | 12 | 0 | 16 | 9 | 26 | 1.860507 |  |  |
| hsa-miR-95 | 0 | 11 | 0 | 16 | 6 | 29 | 1.860507 |  |  |
| hsa-miR-369-5p | 3 | 8 | -1 | 17 | -1 | 36 | 1.862319 |  |  |
| hsa-let-7i | -1 | 12 | -1 | 17 | 11 | 24 | 1.862319 |  |  |
| hsa-miR-885-3p | 0 | 11 | 5 | 11 | -5 | 40 | 1.872283 |  |  |
| hsa-miR-219-1-3p | -1 | 12 | 4 | 12 | 0 | 35 | 1.874094 |  |  |
| hsa-miR-362-5p | 0 | 11 | 4 | 12 | -3 | 38 | 1.874094 |  |  |
| hsa-miR-1977 | 1 | 10 | 2 | 14 | -2 | 37 | 1.877717 |  |  |
| hsa-miR-525-5p | 0 | 11 | 2 | 14 | 1 | 34 | 1.877717 |  |  |
| hsa-miR-1306 | 0 | 11 | 1 | 15 | 3 | 32 | 1.879529 |  |  |
| hsa-miR-516a-5p | 0 | 11 | 1 | 15 | 3 | 32 | 1.879529 |  |  |
| hsa-miR-1286 | 0 | 11 | 1 | 15 | 3 | 32 | 1.879529 |  |  |
| hsa-miR-532-5p | 0 | 11 | 1 | 15 | 3 | 32 | 1.879529 |  |  |
| hsa-miR-381 | 1 | 10 | 1 | 15 | 0 | 35 | 1.879529 |  |  |
| hsa-miR-584 | 1 | 10 | 1 | 15 | 0 | 35 | 1.879529 |  |  |
| hsa-miR-549 | 0 | 11 | 0 | 16 | 5 | 30 | 1.881341 |  |  |
| hsa-miR-378 | 0 | 11 | 0 | 16 | 5 | 30 | 1.881341 |  |  |
| hsa-miR-92b | 0 | 11 | 0 | 16 | 5 | 30 | 1.881341 |  |  |
| hsa-miR-29b | 0 | 11 | 0 | 16 | 5 | 30 | 1.881341 |  |  |
| hsa-let-7g | -1 | 12 | 0 | 16 | 8 | 27 | 1.881341 |  |  |
| hsa-miR-1285 | 2 | 9 | 0 | 16 | -1 | 36 | 1.881341 |  |  |
| hsa-miR-181d | 0 | 11 | 0 | 16 | 5 | 30 | 1.881341 |  |  |
| hsa-miR-628-5p | 0 | 11 | 0 | 16 | 5 | 30 | 1.881341 |  |  |
| hsa-miR-758 | 0 | 11 | 0 | 16 | 5 | 30 | 1.881341 |  |  |
| hsa-miR-891a | 1 | 10 | 0 | 16 | 2 | 33 | 1.881341 |  |  |
| hsa-miR-499-3p | 0 | 11 | 0 | 16 | 5 | 30 | 1.881341 |  |  |
| hsa-miR-1294 | -3 | 14 | -1 | 17 | 16 | 19 | 1.883152 |  |  |
| hsa-miR-181a | 1 | 10 | -2 | 18 | 6 | 29 | 1.884964 |  |  |
| hsa-miR-222 | 0 | 11 | 3 | 13 | -2 | 37 | 1.896739 |  |  |
| hsa-miR-362-3p | 1 | 10 | 3 | 13 | -5 | 40 | 1.896739 |  |  |
| hsa-miR-614 | 0 | 11 | 3 | 13 | -2 | 37 | 1.896739 |  |  |
| hsa-miR-1234 | -3 | 14 | 3 | 13 | 7 | 28 | 1.896739 |  |  |
| hsa-miR-767-3p | 0 | 11 | 2 | 14 | 0 | 35 | 1.898551 |  |  |
| hsa-miR-136 | -1 | 12 | 2 | 14 | 3 | 32 | 1.898551 |  |  |
| hsa-miR-518d-3p | 0 | 11 | 2 | 14 | 0 | 35 | 1.898551 |  |  |
| hsa-miR-1826 | 0 | 11 | 1 | 15 | 2 | 33 | 1.900362 |  |  |
| hsa-miR-943 | 0 | 11 | 1 | 15 | 2 | 33 | 1.900362 |  |  |
| hsa-miR-25 | 0 | 11 | 1 | 15 | 2 | 33 | 1.900362 |  |  |
| hsa-miR-494 | 1 | 10 | 1 | 15 | -1 | 36 | 1.900362 |  |  |
| hsa-miR-371-5p | 0 | 11 | 1 | 15 | 2 | 33 | 1.900362 |  |  |
| hsa-miR-487b | 0 | 11 | 1 | 15 | 2 | 33 | 1.900362 |  |  |
| hsa-miR-551a | 1 | 10 | 1 | 15 | -1 | 36 | 1.900362 |  |  |
| hsa-miR-24 | 0 | 11 | 1 | 15 | 2 | 33 | 1.900362 |  |  |
| hsa-miR-96 | 0 | 11 | 0 | 16 | 4 | 31 | 1.902174 |  |  |
| hsa-miR-576-5p | 0 | 11 | 0 | 16 | 4 | 31 | 1.902174 |  |  |
| hsa-miR-598 | 0 | 11 | 0 | 16 | 4 | 31 | 1.902174 |  |  |
| hsa-miR-15b | 0 | 11 | 0 | 16 | 4 | 31 | 1.902174 |  |  |
| hsa-miR-1271 | 0 | 11 | 0 | 16 | 4 | 31 | 1.902174 |  |  |
| hsa-miR-568 | 1 | 10 | 0 | 16 | 1 | 34 | 1.902174 |  |  |
| hsa-miR-29a | 0 | 11 | 0 | 16 | 4 | 31 | 1.902174 |  |  |
| hsa-miR-93 | 0 | 11 | 0 | 16 | 4 | 31 | 1.902174 |  |  |
| hsa-miR-224 | 0 | 11 | 0 | 16 | 4 | 31 | 1.902174 |  |  |
| hsa-miR-181b | 0 | 11 | 0 | 16 | 4 | 31 | 1.902174 |  |  |
| hsa-miR-616 | -1 | 12 | -1 | 17 | 9 | 26 | 1.903986 |  |  |
| hsa-miR-938 | 1 | 10 | -1 | 17 | 3 | 32 | 1.903986 |  |  |
| hsa-miR-2116 | 1 | 10 | -1 | 17 | 3 | 32 | 1.903986 |  |  |
| hsa-miR-202 | 1 | 10 | -1 | 17 | 3 | 32 | 1.903986 |  |  |
| hsa-miR-129-3p | 0 | 11 | -1 | 17 | 6 | 29 | 1.903986 |  |  |
| hsa-miR-498 | -1 | 12 | -2 | 18 | 11 | 24 | 1.905797 |  |  |
| hsa-miR-198 | -2 | 13 | 5 | 11 | -1 | 36 | 1.913949 |  |  |
| hsa-miR-1183 | 0 | 11 | 3 | 13 | -3 | 38 | 1.917572 |  |  |
| hsa-miR-518b | 0 | 11 | 2 | 14 | -1 | 36 | 1.919384 |  |  |
| hsa-miR-1324 | 0 | 11 | 2 | 14 | -1 | 36 | 1.919384 |  |  |
| hsa-miR-921 | 0 | 11 | 2 | 14 | -1 | 36 | 1.919384 |  |  |
| hsa-miR-106a | 0 | 11 | 2 | 14 | -1 | 36 | 1.919384 |  |  |
| hsa-miR-543 | 0 | 11 | 1 | 15 | 1 | 34 | 1.921196 |  |  |
| hsa-miR-455-3p | 0 | 11 | 1 | 15 | 1 | 34 | 1.921196 |  |  |
| hsa-miR-2277 | 0 | 11 | 1 | 15 | 1 | 34 | 1.921196 |  |  |
| hsa-miR-216a | 0 | 11 | 0 | 16 | 3 | 32 | 1.923007 |  |  |
| hsa-miR-143 | 1 | 10 | 0 | 16 | 0 | 35 | 1.923007 |  |  |
| hsa-miR-449a | 0 | 11 | 0 | 16 | 3 | 32 | 1.923007 |  |  |
| hsa-miR-1252 | 4 | 7 | 0 | 16 | -9 | 44 | 1.923007 |  |  |
| hsa-miR-660 | 0 | 11 | 0 | 16 | 3 | 32 | 1.923007 |  |  |
| hsa-miR-606 | 0 | 11 | 0 | 16 | 3 | 32 | 1.923007 |  |  |
| hsa-miR-421 | 0 | 11 | 0 | 16 | 3 | 32 | 1.923007 |  |  |
| hsa-miR-1243 | 0 | 11 | 0 | 16 | 3 | 32 | 1.923007 |  |  |
| hsa-miR-299-3p | 2 | 9 | 0 | 16 | -3 | 38 | 1.923007 |  |  |
| hsa-miR-1229 | 0 | 11 | 0 | 16 | 3 | 32 | 1.923007 |  |  |
| hsa-miR-620 | 0 | 11 | 0 | 16 | 3 | 32 | 1.923007 |  |  |
| hsa-miR-759 | 0 | 11 | 0 | 16 | 3 | 32 | 1.923007 |  |  |
| hsa-miR-656 | 0 | 11 | 0 | 16 | 3 | 32 | 1.923007 |  |  |
| hsa-miR-577 | 1 | 10 | 0 | 16 | 0 | 35 | 1.923007 |  |  |
| hsa-miR-1298 | 0 | 11 | 0 | 16 | 3 | 32 | 1.923007 |  |  |
| hsa-miR-517c | 0 | 11 | 0 | 16 | 3 | 32 | 1.923007 |  |  |
| hsa-miR-22 | 2 | 9 | -1 | 17 | -1 | 36 | 1.924819 |  |  |
| hsa-miR-2276 | 0 | 11 | -1 | 17 | 5 | 30 | 1.924819 |  |  |
| hsa-miR-103-as | 0 | 11 | -1 | 17 | 5 | 30 | 1.924819 |  |  |
| hsa-miR-1204 | 0 | 11 | -1 | 17 | 5 | 30 | 1.924819 |  |  |
| hsa-miR-886-3p | -2 | 13 | -2 | 18 | 13 | 22 | 1.92663 |  |  |
| hsa-miR-1236 | 0 | 11 | -5 | 21 | 13 | 22 | 1.932065 |  |  |
| hsa-miR-654-5p | 1 | 10 | -7 | 23 | 14 | 21 | 1.935688 |  |  |
| hsa-miR-302c | -1 | 12 | 3 | 13 | -1 | 36 | 1.938406 |  |  |
| hsa-miR-127-5p | 0 | 11 | 2 | 14 | -2 | 37 | 1.940217 |  |  |
| hsa-miR-1304 | -3 | 14 | 1 | 15 | 9 | 26 | 1.942029 |  |  |
| hsa-miR-1267 | 1 | 10 | 1 | 15 | -3 | 38 | 1.942029 |  |  |
| hsa-miR-769-5p | 0 | 11 | 1 | 15 | 0 | 35 | 1.942029 |  |  |
| hsa-miR-652 | 0 | 11 | 1 | 15 | 0 | 35 | 1.942029 |  |  |
| hsa-miR-518a-5p | 1 | 10 | 1 | 15 | -3 | 38 | 1.942029 |  |  |
| hsa-miR-323-3p | 0 | 11 | 1 | 15 | 0 | 35 | 1.942029 |  |  |
| hsa-miR-203 | 1 | 10 | 1 | 15 | -3 | 38 | 1.942029 |  |  |
| hsa-miR-199b-5p | 0 | 11 | 1 | 15 | 0 | 35 | 1.942029 |  |  |
| hsa-miR-151-5p | 2 | 9 | 1 | 15 | -6 | 41 | 1.942029 |  |  |
| hsa-miR-527 | 1 | 10 | 1 | 15 | -3 | 38 | 1.942029 |  |  |
| hsa-miR-485-5p | -1 | 12 | 1 | 15 | 3 | 32 | 1.942029 |  |  |
| hsa-miR-1272 | 0 | 11 | 1 | 15 | 0 | 35 | 1.942029 |  |  |
| hsa-miR-515-3p | 0 | 11 | 1 | 15 | 0 | 35 | 1.942029 |  |  |
| hsa-miR-487a | 0 | 11 | 0 | 16 | 2 | 33 | 1.943841 |  |  |
| hsa-miR-563 | 0 | 11 | 0 | 16 | 2 | 33 | 1.943841 |  |  |
| hsa-miR-1277 | 0 | 11 | 0 | 16 | 2 | 33 | 1.943841 |  |  |
| hsa-miR-586 | 0 | 11 | 0 | 16 | 2 | 33 | 1.943841 |  |  |
| hsa-miR-30b | 0 | 11 | 0 | 16 | 2 | 33 | 1.943841 |  |  |
| hsa-miR-101 | 0 | 11 | 0 | 16 | 2 | 33 | 1.943841 |  |  |
| hsa-miR-7 | 0 | 11 | 0 | 16 | 2 | 33 | 1.943841 |  |  |
| hsa-miR-182 | -1 | 12 | 0 | 16 | 5 | 30 | 1.943841 |  |  |
| hsa-miR-1251 | 0 | 11 | 0 | 16 | 2 | 33 | 1.943841 |  |  |
| hsa-miR-1979 | 0 | 11 | 0 | 16 | 2 | 33 | 1.943841 |  |  |
| hsa-miR-409-3p | 0 | 11 | 0 | 16 | 2 | 33 | 1.943841 |  |  |
| hsa-miR-497 | -1 | 12 | 0 | 16 | 5 | 30 | 1.943841 |  |  |
| hsa-miR-1200 | 2 | 9 | -1 | 17 | -2 | 37 | 1.945652 |  |  |
| hsa-miR-767-5p | 0 | 11 | -2 | 18 | 6 | 29 | 1.947464 |  |  |
| hsa-miR-1178 | 0 | 11 | -4 | 20 | 10 | 25 | 1.951087 |  |  |
| hsa-miR-507 | 0 | 11 | 2 | 14 | -3 | 38 | 1.961051 |  |  |
| hsa-miR-30e | 0 | 11 | 2 | 14 | -3 | 38 | 1.961051 |  |  |
| hsa-miR-191 | 0 | 11 | 1 | 15 | -1 | 36 | 1.962862 |  |  |
| hsa-miR-128 | -1 | 12 | 1 | 15 | 2 | 33 | 1.962862 |  |  |
| hsa-miR-320d | 0 | 11 | 1 | 15 | -1 | 36 | 1.962862 |  |  |
| hsa-miR-516b | 1 | 10 | 1 | 15 | -4 | 39 | 1.962862 |  |  |
| hsa-miR-556-3p | 0 | 11 | 1 | 15 | -1 | 36 | 1.962862 |  |  |
| hsa-miR-627 | 0 | 11 | 1 | 15 | -1 | 36 | 1.962862 |  |  |
| hsa-miR-509-5p | -1 | 12 | 1 | 15 | 2 | 33 | 1.962862 |  |  |
| hsa-miR-140-3p | 0 | 11 | 1 | 15 | -1 | 36 | 1.962862 |  |  |
| hsa-miR-496 | 1 | 10 | 0 | 16 | -2 | 37 | 1.964674 |  |  |
| hsa-miR-548m | 0 | 11 | 0 | 16 | 1 | 34 | 1.964674 |  |  |
| hsa-miR-302e | 0 | 11 | 0 | 16 | 1 | 34 | 1.964674 |  |  |
| hsa-miR-1305 | 0 | 11 | 0 | 16 | 1 | 34 | 1.964674 |  |  |
| hsa-miR-581 | 0 | 11 | 0 | 16 | 1 | 34 | 1.964674 |  |  |
| hsa-miR-552 | 1 | 10 | 0 | 16 | -2 | 37 | 1.964674 |  |  |
| hsa-miR-495 | 0 | 11 | 0 | 16 | 1 | 34 | 1.964674 |  |  |
| hsa-miR-570 | -1 | 12 | 0 | 16 | 4 | 31 | 1.964674 |  |  |
| hsa-miR-607 | 0 | 11 | 0 | 16 | 1 | 34 | 1.964674 |  |  |
| hsa-miR-629 | -1 | 12 | 0 | 16 | 4 | 31 | 1.964674 |  |  |
| hsa-miR-1206 | 0 | 11 | 0 | 16 | 1 | 34 | 1.964674 |  |  |
| hsa-miR-934 | 0 | 11 | 0 | 16 | 1 | 34 | 1.964674 |  |  |
| hsa-miR-523 | -1 | 12 | 0 | 16 | 4 | 31 | 1.964674 |  |  |
| hsa-miR-889 | 0 | 11 | 0 | 16 | 1 | 34 | 1.964674 |  |  |
| hsa-miR-556-5p | 0 | 11 | 0 | 16 | 1 | 34 | 1.964674 |  |  |
| hsa-miR-640 | 0 | 11 | 0 | 16 | 1 | 34 | 1.964674 |  |  |
| hsa-miR-1278 | 0 | 11 | 0 | 16 | 1 | 34 | 1.964674 |  |  |
| hsa-miR-106b | 0 | 11 | 0 | 16 | 1 | 34 | 1.964674 |  |  |
| hsa-miR-26a | 0 | 11 | 0 | 16 | 1 | 34 | 1.964674 |  |  |
| hsa-miR-891b | 0 | 11 | 0 | 16 | 1 | 34 | 1.964674 |  |  |
| hsa-miR-1281 | -1 | 12 | -1 | 17 | 6 | 29 | 1.966486 |  |  |
| hsa-miR-146a | 2 | 9 | -1 | 17 | -3 | 38 | 1.966486 |  |  |
| hsa-miR-578 | 0 | 11 | -1 | 17 | 3 | 32 | 1.966486 |  |  |
| hsa-miR-517a | 0 | 11 | -2 | 18 | 5 | 30 | 1.968297 |  |  |
| hsa-miR-643 | 0 | 11 | -2 | 18 | 5 | 30 | 1.968297 |  |  |
| hsa-miR-512-3p | -2 | 13 | 4 | 12 | -2 | 37 | 1.978261 |  |  |
| hsa-miR-146b-3p | 1 | 10 | 2 | 14 | -7 | 42 | 1.981884 |  |  |
| hsa-miR-376b | 0 | 11 | 1 | 15 | -2 | 37 | 1.983696 |  |  |
| hsa-miR-502-3p | -1 | 12 | 1 | 15 | 1 | 34 | 1.983696 |  |  |
| hsa-miR-133a | 0 | 11 | 1 | 15 | -2 | 37 | 1.983696 |  |  |
| hsa-miR-532-3p | 0 | 11 | 1 | 15 | -2 | 37 | 1.983696 |  |  |
| hsa-miR-30a | 0 | 11 | 1 | 15 | -2 | 37 | 1.983696 |  |  |
| hsa-miR-548o | -1 | 12 | 1 | 15 | 1 | 34 | 1.983696 |  |  |
| hsa-miR-654-3p | 1 | 10 | 1 | 15 | -5 | 40 | 1.983696 |  |  |
| hsa-miR-301a | 0 | 11 | 1 | 15 | -2 | 37 | 1.983696 |  |  |
| hsa-miR-583 | 0 | 11 | 1 | 15 | -2 | 37 | 1.983696 |  |  |
| hsa-miR-671-5p | 0 | 11 | 1 | 15 | -2 | 37 | 1.983696 |  |  |
| hsa-miR-380 | 0 | 11 | 0 | 16 | 0 | 35 | 1.985507 |  |  |
| hsa-miR-875-5p | 0 | 11 | 0 | 16 | 0 | 35 | 1.985507 |  |  |
| hsa-miR-192 | 0 | 11 | 0 | 16 | 0 | 35 | 1.985507 |  |  |
| hsa-miR-374b | 0 | 11 | 0 | 16 | 0 | 35 | 1.985507 |  |  |
| hsa-miR-125a-5p | 0 | 11 | 0 | 16 | 0 | 35 | 1.985507 |  |  |
| hsa-miR-129-5p | 0 | 11 | 0 | 16 | 0 | 35 | 1.985507 |  |  |
| hsa-miR-342-3p | 1 | 10 | 0 | 16 | -3 | 38 | 1.985507 |  |  |
| hsa-miR-651 | 0 | 11 | 0 | 16 | 0 | 35 | 1.985507 |  |  |
| hsa-miR-23a | 0 | 11 | 0 | 16 | 0 | 35 | 1.985507 |  |  |
| hsa-miR-302f | 0 | 11 | 0 | 16 | 0 | 35 | 1.985507 |  |  |
| hsa-miR-548f | 0 | 11 | 0 | 16 | 0 | 35 | 1.985507 |  |  |
| hsa-miR-29c | 0 | 11 | 0 | 16 | 0 | 35 | 1.985507 |  |  |
| hsa-miR-132 | 0 | 11 | 0 | 16 | 0 | 35 | 1.985507 |  |  |
| hsa-miR-1297 | 0 | 11 | 0 | 16 | 0 | 35 | 1.985507 |  |  |
| hsa-miR-579 | 0 | 11 | 0 | 16 | 0 | 35 | 1.985507 |  |  |
| hsa-miR-384 | 0 | 11 | 0 | 16 | 0 | 35 | 1.985507 |  |  |
| hsa-miR-429 | -1 | 12 | 0 | 16 | 3 | 32 | 1.985507 |  |  |
| hsa-miR-588 | -1 | 12 | 0 | 16 | 3 | 32 | 1.985507 |  |  |
| hsa-miR-1258 | 0 | 11 | 0 | 16 | 0 | 35 | 1.985507 |  |  |
| hsa-miR-1274a | 0 | 11 | 0 | 16 | 0 | 35 | 1.985507 |  |  |
| hsa-miR-539 | 0 | 11 | 0 | 16 | 0 | 35 | 1.985507 |  |  |
| hsa-miR-98 | -1 | 12 | 0 | 16 | 3 | 32 | 1.985507 |  |  |
| hsa-miR-1185 | 0 | 11 | 0 | 16 | 0 | 35 | 1.985507 |  |  |
| hsa-miR-2053 | 0 | 11 | 0 | 16 | 0 | 35 | 1.985507 |  |  |
| hsa-miR-219-5p | 0 | 11 | 0 | 16 | 0 | 35 | 1.985507 |  |  |
| hsa-miR-551b | 0 | 11 | 0 | 16 | 0 | 35 | 1.985507 |  |  |
| hsa-miR-574-3p | 0 | 11 | -1 | 17 | 2 | 33 | 1.987319 |  |  |
| hsa-miR-1201 | 1 | 10 | -1 | 17 | -1 | 36 | 1.987319 |  |  |
| hsa-miR-26b | 0 | 11 | -1 | 17 | 2 | 33 | 1.987319 |  |  |
| hsa-miR-34a | 0 | 11 | -1 | 17 | 2 | 33 | 1.987319 |  |  |
| hsa-miR-412 | 0 | 11 | -1 | 17 | 2 | 33 | 1.987319 |  |  |
| hsa-miR-137 | 0 | 11 | -1 | 17 | 2 | 33 | 1.987319 |  |  |
| hsa-miR-300 | 0 | 11 | -1 | 17 | 2 | 33 | 1.987319 |  |  |
| hsa-miR-485-3p | 0 | 11 | -1 | 17 | 2 | 33 | 1.987319 |  |  |
| hsa-miR-1197 | -1 | 12 | -1 | 17 | 5 | 30 | 1.987319 |  |  |
| hsa-miR-646 | 0 | 11 | -2 | 18 | 4 | 31 | 1.98913 |  |  |
| hsa-miR-220c | -1 | 12 | -2 | 18 | 7 | 28 | 1.98913 |  |  |
| hsa-miR-181c | 0 | 11 | -2 | 18 | 4 | 31 | 1.98913 |  |  |
| hsa-miR-107 | 0 | 11 | -2 | 18 | 4 | 31 | 1.98913 |  |  |
| hsa-miR-587 | -1 | 12 | 1 | 15 | 0 | 35 | 2.004529 |  |  |
| hsa-miR-572 | -1 | 12 | 1 | 15 | 0 | 35 | 2.004529 |  |  |
| hsa-miR-492 | 0 | 11 | 1 | 15 | -3 | 38 | 2.004529 |  |  |
| hsa-miR-562 | -1 | 12 | 0 | 16 | 2 | 33 | 2.006341 |  |  |
| hsa-miR-548g | 0 | 11 | 0 | 16 | -1 | 36 | 2.006341 |  |  |
| hsa-miR-1179 | 0 | 11 | 0 | 16 | -1 | 36 | 2.006341 |  |  |
| hsa-miR-603 | -1 | 12 | 0 | 16 | 2 | 33 | 2.006341 |  |  |
| hsa-miR-208a | 0 | 11 | 0 | 16 | -1 | 36 | 2.006341 |  |  |
| hsa-miR-186 | 0 | 11 | 0 | 16 | -1 | 36 | 2.006341 |  |  |
| hsa-miR-1227 | -1 | 12 | 0 | 16 | 2 | 33 | 2.006341 |  |  |
| hsa-miR-569 | 0 | 11 | 0 | 16 | -1 | 36 | 2.006341 |  |  |
| hsa-miR-450a | 1 | 10 | 0 | 16 | -4 | 39 | 2.006341 |  |  |
| hsa-miR-624 | 0 | 11 | 0 | 16 | -1 | 36 | 2.006341 |  |  |
| hsa-miR-1208 | 0 | 11 | 0 | 16 | -1 | 36 | 2.006341 |  |  |
| hsa-miR-888 | 0 | 11 | 0 | 16 | -1 | 36 | 2.006341 |  |  |
| hsa-miR-105 | 0 | 11 | 0 | 16 | -1 | 36 | 2.006341 |  |  |
| hsa-miR-124 | 0 | 11 | 0 | 16 | -1 | 36 | 2.006341 |  |  |
| hsa-miR-1261 | -1 | 12 | 0 | 16 | 2 | 33 | 2.006341 |  |  |
| hsa-miR-554 | 0 | 11 | 0 | 16 | -1 | 36 | 2.006341 |  |  |
| hsa-miR-2052 | 0 | 11 | 0 | 16 | -1 | 36 | 2.006341 |  |  |
| hsa-miR-99b | 0 | 11 | 0 | 16 | -1 | 36 | 2.006341 |  |  |
| hsa-miR-520h | 0 | 11 | 0 | 16 | -1 | 36 | 2.006341 |  |  |
| hsa-miR-555 | 0 | 11 | 0 | 16 | -1 | 36 | 2.006341 |  |  |
| hsa-miR-297 | 0 | 11 | 0 | 16 | -1 | 36 | 2.006341 |  |  |
| hsa-miR-374a | 0 | 11 | 0 | 16 | -1 | 36 | 2.006341 |  |  |
| hsa-miR-1537 | 0 | 11 | 0 | 16 | -1 | 36 | 2.006341 |  |  |
| hsa-miR-876-3p | -2 | 13 | 0 | 16 | 5 | 30 | 2.006341 |  |  |
| hsa-miR-944 | 0 | 11 | 0 | 16 | -1 | 36 | 2.006341 |  |  |
| hsa-miR-613 | 0 | 11 | 0 | 16 | -1 | 36 | 2.006341 |  |  |
| hsa-miR-299-5p | 0 | 11 | -1 | 17 | 1 | 34 | 2.008152 |  |  |
| hsa-miR-338-5p | 0 | 11 | -1 | 17 | 1 | 34 | 2.008152 |  |  |
| hsa-miR-664 | 0 | 11 | -1 | 17 | 1 | 34 | 2.008152 |  |  |
| hsa-miR-761 | 1 | 10 | -1 | 17 | -2 | 37 | 2.008152 |  |  |
| hsa-miR-1226 | -1 | 12 | -1 | 17 | 4 | 31 | 2.008152 |  |  |
| hsa-miR-125a-3p | -5 | 16 | -1 | 17 | 16 | 19 | 2.008152 |  |  |
| hsa-miR-634 | 0 | 11 | -1 | 17 | 1 | 34 | 2.008152 |  |  |
| hsa-miR-1284 | 0 | 11 | -1 | 17 | 1 | 34 | 2.008152 |  |  |
| hsa-miR-483-3p | -2 | 13 | -2 | 18 | 9 | 26 | 2.009964 |  |  |
| hsa-miR-431 | 0 | 11 | -2 | 18 | 3 | 32 | 2.009964 |  |  |
| hsa-miR-648 | 0 | 11 | -3 | 19 | 5 | 30 | 2.011775 |  |  |
| hsa-miR-519b-5p | -1 | 12 | -3 | 19 | 8 | 27 | 2.011775 |  |  |
| hsa-miR-519c-5p | -1 | 12 | -3 | 19 | 8 | 27 | 2.011775 |  |  |
| hsa-miR-155 | 0 | 11 | 2 | 14 | -6 | 41 | 2.023551 |  |  |
| hsa-miR-493 | 0 | 11 | 2 | 14 | -6 | 41 | 2.023551 |  |  |
| hsa-miR-199a-3p | -1 | 12 | 2 | 14 | -3 | 38 | 2.023551 |  |  |
| hsa-miR-199b-3p | -1 | 12 | 2 | 14 | -3 | 38 | 2.023551 |  |  |
| hsa-miR-561 | 0 | 11 | 1 | 15 | -4 | 39 | 2.025362 |  |  |
| hsa-miR-30d | -1 | 12 | 1 | 15 | -1 | 36 | 2.025362 |  |  |
| hsa-miR-2278 | 0 | 11 | 0 | 16 | -2 | 37 | 2.027174 |  |  |
| hsa-miR-559 | 0 | 11 | 0 | 16 | -2 | 37 | 2.027174 |  |  |
| hsa-miR-99a | 0 | 11 | 0 | 16 | -2 | 37 | 2.027174 |  |  |
| hsa-miR-544 | 0 | 11 | 0 | 16 | -2 | 37 | 2.027174 |  |  |
| hsa-miR-519a | 0 | 11 | 0 | 16 | -2 | 37 | 2.027174 |  |  |
| hsa-miR-590-5p | 0 | 11 | 0 | 16 | -2 | 37 | 2.027174 |  |  |
| hsa-miR-2113 | 1 | 10 | 0 | 16 | -5 | 40 | 2.027174 |  |  |
| hsa-miR-599 | 0 | 11 | 0 | 16 | -2 | 37 | 2.027174 |  |  |
| hsa-miR-215 | 0 | 11 | 0 | 16 | -2 | 37 | 2.027174 |  |  |
| hsa-miR-144 | 0 | 11 | 0 | 16 | -2 | 37 | 2.027174 |  |  |
| hsa-miR-633 | 0 | 11 | 0 | 16 | -2 | 37 | 2.027174 |  |  |
| hsa-miR-653 | 0 | 11 | 0 | 16 | -2 | 37 | 2.027174 |  |  |
| hsa-miR-126 | 0 | 11 | 0 | 16 | -2 | 37 | 2.027174 |  |  |
| hsa-miR-545 | 0 | 11 | 0 | 16 | -2 | 37 | 2.027174 |  |  |
| hsa-miR-514 | 0 | 11 | 0 | 16 | -2 | 37 | 2.027174 |  |  |
| hsa-miR-491-3p | 0 | 11 | 0 | 16 | -2 | 37 | 2.027174 |  |  |
| hsa-miR-1322 | 0 | 11 | 0 | 16 | -2 | 37 | 2.027174 |  |  |
| hsa-miR-548a-3p | 0 | 11 | 0 | 16 | -2 | 37 | 2.027174 |  |  |
| hsa-miR-597 | -1 | 12 | 0 | 16 | 1 | 34 | 2.027174 |  |  |
| hsa-miR-1827 | 0 | 11 | 0 | 16 | -2 | 37 | 2.027174 |  |  |
| hsa-miR-1264 | 0 | 11 | 0 | 16 | -2 | 37 | 2.027174 |  |  |
| hsa-miR-1274b | -1 | 12 | 0 | 16 | 1 | 34 | 2.027174 |  |  |
| hsa-miR-626 | -1 | 12 | 0 | 16 | 1 | 34 | 2.027174 |  |  |
| hsa-miR-499-5p | 0 | 11 | 0 | 16 | -2 | 37 | 2.027174 |  |  |
| hsa-miR-1302 | -1 | 12 | 0 | 16 | 1 | 34 | 2.027174 |  |  |
| hsa-miR-558 | 0 | 11 | 0 | 16 | -2 | 37 | 2.027174 |  |  |
| hsa-miR-618 | -1 | 12 | -1 | 17 | 3 | 32 | 2.028986 |  |  |
| hsa-miR-610 | -1 | 12 | -1 | 17 | 3 | 32 | 2.028986 |  |  |
| hsa-miR-609 | -1 | 12 | -1 | 17 | 3 | 32 | 2.028986 |  |  |
| hsa-miR-522 | 0 | 11 | -1 | 17 | 0 | 35 | 2.028986 |  |  |
| hsa-miR-1283 | 0 | 11 | -1 | 17 | 0 | 35 | 2.028986 |  |  |
| hsa-miR-591 | 0 | 11 | -2 | 18 | 2 | 33 | 2.030797 |  |  |
| hsa-miR-596 | -1 | 12 | -2 | 18 | 5 | 30 | 2.030797 |  |  |
| hsa-miR-519d | 0 | 11 | -2 | 18 | 2 | 33 | 2.030797 |  |  |
| hsa-miR-1184 | -1 | 12 | -3 | 19 | 7 | 28 | 2.032609 |  |  |
| hsa-miR-668 | 0 | 11 | -3 | 19 | 4 | 31 | 2.032609 |  |  |
| hsa-miR-148a | 0 | 11 | -3 | 19 | 4 | 31 | 2.032609 |  |  |
| hsa-miR-452 | 0 | 11 | 4 | 12 | -11 | 46 | 2.040761 |  |  |
| hsa-miR-130b | -1 | 12 | 3 | 13 | -6 | 41 | 2.042572 |  |  |
| hsa-miR-524-3p | 0 | 11 | 2 | 14 | -7 | 42 | 2.044384 |  |  |
| hsa-miR-575 | 1 | 10 | 1 | 15 | -8 | 43 | 2.046196 |  |  |
| hsa-miR-521 | 0 | 11 | 1 | 15 | -5 | 40 | 2.046196 |  |  |
| hsa-miR-628-3p | 0 | 11 | 1 | 15 | -5 | 40 | 2.046196 |  |  |
| hsa-miR-17 | 0 | 11 | 1 | 15 | -5 | 40 | 2.046196 |  |  |
| hsa-miR-190 | 0 | 11 | 0 | 16 | -3 | 38 | 2.048007 |  |  |
| hsa-miR-548p | 0 | 11 | 0 | 16 | -3 | 38 | 2.048007 |  |  |
| hsa-miR-582-5p | 0 | 11 | 0 | 16 | -3 | 38 | 2.048007 |  |  |
| hsa-miR-301b | 0 | 11 | 0 | 16 | -3 | 38 | 2.048007 |  |  |
| hsa-miR-10a | 0 | 11 | 0 | 16 | -3 | 38 | 2.048007 |  |  |
| hsa-miR-411 | 0 | 11 | 0 | 16 | -3 | 38 | 2.048007 |  |  |
| hsa-miR-590-3p | 0 | 11 | 0 | 16 | -3 | 38 | 2.048007 |  |  |
| hsa-miR-553 | 0 | 11 | 0 | 16 | -3 | 38 | 2.048007 |  |  |
| hsa-miR-802 | 0 | 11 | 0 | 16 | -3 | 38 | 2.048007 |  |  |
| hsa-miR-488 | 1 | 10 | 0 | 16 | -6 | 41 | 2.048007 |  |  |
| hsa-miR-450b-5p | 0 | 11 | 0 | 16 | -3 | 38 | 2.048007 |  |  |
| hsa-miR-135b | 0 | 11 | 0 | 16 | -3 | 38 | 2.048007 |  |  |
| hsa-miR-27b | 0 | 11 | 0 | 16 | -3 | 38 | 2.048007 |  |  |
| hsa-miR-33b | -1 | 12 | 0 | 16 | 0 | 35 | 2.048007 |  |  |
| hsa-miR-141 | 0 | 11 | 0 | 16 | -3 | 38 | 2.048007 |  |  |
| hsa-miR-1978 | 0 | 11 | 0 | 16 | -3 | 38 | 2.048007 |  |  |
| hsa-miR-208b | 0 | 11 | 0 | 16 | -3 | 38 | 2.048007 |  |  |
| hsa-miR-548c-3p | 0 | 11 | 0 | 16 | -3 | 38 | 2.048007 |  |  |
| hsa-miR-454 | 0 | 11 | 0 | 16 | -3 | 38 | 2.048007 |  |  |
| hsa-miR-548k | 0 | 11 | 0 | 16 | -3 | 38 | 2.048007 |  |  |
| hsa-miR-655 | -1 | 12 | 0 | 16 | 0 | 35 | 2.048007 |  |  |
| hsa-miR-924 | 0 | 11 | 0 | 16 | -3 | 38 | 2.048007 |  |  |
| hsa-miR-1973 | 0 | 11 | 0 | 16 | -3 | 38 | 2.048007 |  |  |
| hsa-miR-2054 | 0 | 11 | 0 | 16 | -3 | 38 | 2.048007 |  |  |
| hsa-miR-218 | 0 | 11 | 0 | 16 | -3 | 38 | 2.048007 |  |  |
| hsa-miR-142-3p | 0 | 11 | 0 | 16 | -3 | 38 | 2.048007 |  |  |
| hsa-miR-2115 | 0 | 11 | -1 | 17 | -1 | 36 | 2.049819 |  |  |
| hsa-miR-525-3p | 1 | 10 | -1 | 17 | -4 | 39 | 2.049819 |  |  |
| hsa-miR-21 | 0 | 11 | -1 | 17 | -1 | 36 | 2.049819 |  |  |
| hsa-miR-187 | 0 | 11 | -2 | 18 | 1 | 34 | 2.05163 |  |  |
| hsa-miR-520g | 0 | 11 | -2 | 18 | 1 | 34 | 2.05163 |  |  |
| hsa-miR-337-5p | 0 | 11 | -2 | 18 | 1 | 34 | 2.05163 |  |  |
| hsa-miR-1205 | 1 | 10 | -3 | 19 | 0 | 35 | 2.053442 |  |  |
| hsa-miR-1975 | 1 | 10 | -3 | 19 | 0 | 35 | 2.053442 |  |  |
| hsa-miR-518a-3p | 0 | 11 | 2 | 14 | -8 | 43 | 2.065217 |  |  |
| hsa-miR-515-5p | 0 | 11 | 2 | 14 | -8 | 43 | 2.065217 |  |  |
| hsa-miR-214 | 1 | 10 | 1 | 15 | -9 | 44 | 2.067029 |  |  |
| hsa-miR-377 | 0 | 11 | 1 | 15 | -6 | 41 | 2.067029 |  |  |
| hsa-miR-1280 | -2 | 13 | 1 | 15 | 0 | 35 | 2.067029 |  |  |
| hsa-miR-361-5p | -1 | 12 | 1 | 15 | -3 | 38 | 2.067029 |  |  |
| hsa-let-7c | -3 | 14 | 1 | 15 | 3 | 32 | 2.067029 |  |  |
| hsa-miR-142-5p | -1 | 12 | 0 | 16 | -1 | 36 | 2.068841 |  |  |
| hsa-miR-194 | 0 | 11 | 0 | 16 | -4 | 39 | 2.068841 |  |  |
| hsa-miR-1912 | 0 | 11 | 0 | 16 | -4 | 39 | 2.068841 |  |  |
| hsa-miR-34c-3p | -1 | 12 | 0 | 16 | -1 | 36 | 2.068841 |  |  |
| hsa-miR-1259 | 0 | 11 | 0 | 16 | -4 | 39 | 2.068841 |  |  |
| hsa-miR-448 | 1 | 10 | -1 | 17 | -5 | 40 | 2.070652 |  |  |
| hsa-miR-410 | -1 | 12 | -1 | 17 | 1 | 34 | 2.070652 |  |  |
| hsa-miR-200a | 0 | 11 | -1 | 17 | -2 | 37 | 2.070652 |  |  |
| hsa-miR-152 | 0 | 11 | -1 | 17 | -2 | 37 | 2.070652 |  |  |
| hsa-miR-1256 | -1 | 12 | -1 | 17 | 1 | 34 | 2.070652 |  |  |
| hsa-miR-1279 | -1 | 12 | -1 | 17 | 1 | 34 | 2.070652 |  |  |
| hsa-miR-622 | 0 | 11 | -2 | 18 | 0 | 35 | 2.072464 |  |  |
| hsa-miR-1224-3p | -1 | 12 | -2 | 18 | 3 | 32 | 2.072464 |  |  |
| hsa-miR-892a | 0 | 11 | -2 | 18 | 0 | 35 | 2.072464 |  |  |
| hsa-miR-330-3p | -1 | 12 | -3 | 19 | 5 | 30 | 2.074275 |  |  |
| hsa-miR-585 | 0 | 11 | 1 | 15 | -7 | 42 | 2.087862 |  |  |
| hsa-miR-31 | 0 | 11 | 1 | 15 | -7 | 42 | 2.087862 |  |  |
| hsa-miR-296-5p | -1 | 12 | 1 | 15 | -4 | 39 | 2.087862 |  |  |
| hsa-miR-223 | -1 | 12 | 1 | 15 | -4 | 39 | 2.087862 |  |  |
| hsa-miR-449c | 0 | 11 | 1 | 15 | -7 | 42 | 2.087862 |  |  |
| hsa-miR-548l | -1 | 12 | 1 | 15 | -4 | 39 | 2.087862 |  |  |
| hsa-miR-509-3-5p | 0 | 11 | 0 | 16 | -5 | 40 | 2.089674 |  |  |
| hsa-let-7d | -3 | 14 | 0 | 16 | 4 | 31 | 2.089674 |  |  |
| hsa-miR-890 | -1 | 12 | 0 | 16 | -2 | 37 | 2.089674 |  |  |
| hsa-miR-548a-5p | 0 | 11 | 0 | 16 | -5 | 40 | 2.089674 |  |  |
| hsa-miR-376c | 0 | 11 | 0 | 16 | -5 | 40 | 2.089674 |  |  |
| hsa-miR-27a | 0 | 11 | 0 | 16 | -5 | 40 | 2.089674 |  |  |
| hsa-miR-548n | 0 | 11 | 0 | 16 | -5 | 40 | 2.089674 |  |  |
| hsa-miR-1 | 0 | 11 | 0 | 16 | -5 | 40 | 2.089674 |  |  |
| hsa-miR-376a | 0 | 11 | 0 | 16 | -5 | 40 | 2.089674 |  |  |
| hsa-miR-2117 | -1 | 12 | 0 | 16 | -2 | 37 | 2.089674 |  |  |
| hsa-miR-548j | 0 | 11 | 0 | 16 | -5 | 40 | 2.089674 |  |  |
| hsa-miR-424 | 0 | 11 | 0 | 16 | -5 | 40 | 2.089674 |  |  |
| hsa-miR-15a | 0 | 11 | 0 | 16 | -5 | 40 | 2.089674 |  |  |
| hsa-miR-516a-3p | 0 | 11 | -1 | 17 | -3 | 38 | 2.091486 |  |  |
| hsa-miR-337-3p | 0 | 11 | -1 | 17 | -3 | 38 | 2.091486 |  |  |
| hsa-miR-139-5p | 0 | 11 | -1 | 17 | -3 | 38 | 2.091486 |  |  |
| hsa-miR-1265 | -1 | 12 | -1 | 17 | 0 | 35 | 2.091486 |  |  |
| hsa-miR-513a-3p | 0 | 11 | -2 | 18 | -1 | 36 | 2.093297 |  |  |
| hsa-miR-339-3p | 1 | 10 | -2 | 18 | -4 | 39 | 2.093297 |  |  |
| hsa-miR-200c | 0 | 11 | -2 | 18 | -1 | 36 | 2.093297 |  |  |
| hsa-miR-1181 | 0 | 11 | -2 | 18 | -1 | 36 | 2.093297 |  |  |
| hsa-miR-548b-3p | 0 | 11 | -2 | 18 | -1 | 36 | 2.093297 |  |  |
| hsa-miR-503 | 0 | 11 | -3 | 19 | 1 | 34 | 2.095109 |  |  |
| hsa-miR-298 | -1 | 12 | 4 | 12 | -11 | 46 | 2.103261 |  |  |
| hsa-miR-920 | -1 | 12 | 2 | 14 | -7 | 42 | 2.106884 |  |  |
| hsa-miR-518c | 0 | 11 | 1 | 15 | -8 | 43 | 2.108696 |  |  |
| hsa-miR-1539 | -1 | 12 | 1 | 15 | -5 | 40 | 2.108696 |  |  |
| hsa-miR-32 | 0 | 11 | 0 | 16 | -6 | 41 | 2.110507 |  |  |
| hsa-miR-16 | 0 | 11 | 0 | 16 | -6 | 41 | 2.110507 |  |  |
| hsa-miR-19b | 0 | 11 | 0 | 16 | -6 | 41 | 2.110507 |  |  |
| hsa-miR-153 | 0 | 11 | 0 | 16 | -6 | 41 | 2.110507 |  |  |
| hsa-miR-519e | -1 | 12 | 0 | 16 | -3 | 38 | 2.110507 |  |  |
| hsa-miR-216b | 0 | 11 | -1 | 17 | -4 | 39 | 2.112319 |  |  |
| hsa-miR-548d-3p | 0 | 11 | -1 | 17 | -4 | 39 | 2.112319 |  |  |
| hsa-let-7f | -2 | 13 | -1 | 17 | 2 | 33 | 2.112319 |  |  |
| hsa-miR-617 | 1 | 10 | -1 | 17 | -7 | 42 | 2.112319 |  |  |
| hsa-miR-147 | -1 | 12 | -1 | 17 | -1 | 36 | 2.112319 |  |  |
| hsa-miR-382 | 1 | 10 | -1 | 17 | -7 | 42 | 2.112319 |  |  |
| hsa-miR-363 | 0 | 11 | -1 | 17 | -4 | 39 | 2.112319 |  |  |
| hsa-miR-148b | 0 | 11 | -2 | 18 | -2 | 37 | 2.11413 |  |  |
| hsa-miR-589 | 0 | 11 | -2 | 18 | -2 | 37 | 2.11413 |  |  |
| hsa-miR-720 | 0 | 11 | -3 | 19 | 0 | 35 | 2.115942 |  |  |
| hsa-miR-520f | 1 | 10 | -3 | 19 | -3 | 38 | 2.115942 |  |  |
| hsa-miR-30c | 0 | 11 | -3 | 19 | 0 | 35 | 2.115942 |  |  |
| hsa-miR-513b | 0 | 11 | 1 | 15 | -9 | 44 | 2.129529 |  |  |
| hsa-miR-379 | 0 | 11 | 1 | 15 | -9 | 44 | 2.129529 |  |  |
| hsa-miR-600 | 0 | 11 | 1 | 15 | -9 | 44 | 2.129529 |  |  |
| hsa-miR-135a | 0 | 11 | 1 | 15 | -9 | 44 | 2.129529 |  |  |
| hsa-miR-644 | 0 | 11 | 0 | 16 | -7 | 42 | 2.131341 |  |  |
| hsa-miR-876-5p | -1 | 12 | 0 | 16 | -4 | 39 | 2.131341 |  |  |
| hsa-miR-641 | -1 | 12 | 0 | 16 | -4 | 39 | 2.131341 |  |  |
| hsa-miR-592 | -1 | 12 | 0 | 16 | -4 | 39 | 2.131341 |  |  |
| hsa-miR-10b | 0 | 11 | 0 | 16 | -7 | 42 | 2.131341 |  |  |
| hsa-miR-302b | -1 | 12 | 0 | 16 | -4 | 39 | 2.131341 |  |  |
| hsa-miR-548e | 0 | 11 | 0 | 16 | -7 | 42 | 2.131341 |  |  |
| hsa-miR-649 | -1 | 12 | 0 | 16 | -4 | 39 | 2.131341 |  |  |
| hsa-miR-506 | 1 | 10 | -1 | 17 | -8 | 43 | 2.133152 |  |  |
| hsa-miR-550 | -3 | 14 | 3 | 13 | -5 | 40 | 2.146739 |  |  |
| hsa-miR-922 | -1 | 12 | 1 | 15 | -7 | 42 | 2.150362 |  |  |
| hsa-miR-320c | -2 | 13 | 1 | 15 | -4 | 39 | 2.150362 |  |  |
| hsa-miR-20a | 0 | 11 | 1 | 15 | -10 | 45 | 2.150362 |  |  |
| hsa-miR-335 | 0 | 11 | 0 | 16 | -8 | 43 | 2.152174 |  |  |
| hsa-miR-367 | 0 | 11 | 0 | 16 | -8 | 43 | 2.152174 |  |  |
| hsa-miR-548i | 0 | 11 | 0 | 16 | -8 | 43 | 2.152174 |  |  |
| hsa-miR-582-3p | 0 | 11 | 0 | 16 | -8 | 43 | 2.152174 |  |  |
| hsa-miR-190b | 0 | 11 | 0 | 16 | -8 | 43 | 2.152174 |  |  |
| hsa-miR-548d-5p | 0 | 11 | 0 | 16 | -8 | 43 | 2.152174 |  |  |
| hsa-miR-548h | -1 | 12 | 0 | 16 | -5 | 40 | 2.152174 |  |  |
| hsa-miR-33a | -1 | 12 | 0 | 16 | -5 | 40 | 2.152174 |  |  |
| hsa-miR-340 | 0 | 11 | 0 | 16 | -8 | 43 | 2.152174 |  |  |
| hsa-miR-369-3p | -1 | 12 | 0 | 16 | -5 | 40 | 2.152174 |  |  |
| hsa-miR-34b | -3 | 14 | -1 | 17 | 3 | 32 | 2.153986 |  |  |
| hsa-miR-196a | -1 | 12 | -1 | 17 | -3 | 38 | 2.153986 |  |  |
| hsa-miR-371-3p | 0 | 11 | -2 | 18 | -4 | 39 | 2.155797 |  |  |
| hsa-miR-1911 | 0 | 11 | -3 | 19 | -2 | 37 | 2.157609 |  |  |
| hsa-miR-139-3p | -2 | 13 | 2 | 14 | -7 | 42 | 2.169384 |  |  |
| hsa-miR-877 | -1 | 12 | 0 | 16 | -6 | 41 | 2.173007 |  |  |
| hsa-miR-542-3p | -1 | 12 | 0 | 16 | -6 | 41 | 2.173007 |  |  |
| hsa-miR-519c-3p | 0 | 11 | 0 | 16 | -9 | 44 | 2.173007 |  |  |
| hsa-miR-302d | -1 | 12 | 0 | 16 | -6 | 41 | 2.173007 |  |  |
| hsa-miR-34c-5p | 0 | 11 | -1 | 17 | -7 | 42 | 2.174819 |  |  |
| hsa-miR-630 | -2 | 13 | -1 | 17 | -1 | 36 | 2.174819 |  |  |
| hsa-miR-708 | 0 | 11 | -1 | 17 | -7 | 42 | 2.174819 |  |  |
| hsa-miR-520e | -1 | 12 | -1 | 17 | -4 | 39 | 2.174819 |  |  |
| hsa-miR-133b | 0 | 11 | -3 | 19 | -3 | 38 | 2.178442 |  |  |
| hsa-miR-573 | -1 | 12 | 0 | 16 | -7 | 42 | 2.193841 |  |  |
| hsa-miR-195 | 0 | 11 | 0 | 16 | -10 | 45 | 2.193841 |  |  |
| hsa-let-7a | -3 | 14 | 0 | 16 | -1 | 36 | 2.193841 |  |  |
| hsa-miR-19a | -1 | 12 | 0 | 16 | -7 | 42 | 2.193841 |  |  |
| hsa-miR-510 | -2 | 13 | -1 | 17 | -2 | 37 | 2.195652 |  |  |
| hsa-miR-764 | -3 | 14 | -1 | 17 | 1 | 34 | 2.195652 |  |  |
| hsa-miR-875-3p | 0 | 11 | -2 | 18 | -6 | 41 | 2.197464 |  |  |
| hsa-miR-567 | 0 | 11 | -2 | 18 | -6 | 41 | 2.197464 |  |  |
| hsa-miR-220b | 0 | 11 | -3 | 19 | -4 | 39 | 2.199275 |  |  |
| hsa-miR-766 | 1 | 10 | -6 | 22 | -1 | 36 | 2.20471 |  |  |
| hsa-miR-1246 | -3 | 14 | 1 | 15 | -4 | 39 | 2.212862 |  |  |
| hsa-miR-320a | -1 | 12 | 1 | 15 | -10 | 45 | 2.212862 |  |  |
| hsa-miR-576-3p | 0 | 11 | 0 | 16 | -11 | 46 | 2.214674 |  |  |
| hsa-miR-519b-3p | -1 | 12 | 0 | 16 | -8 | 43 | 2.214674 |  |  |
| hsa-miR-103 | 0 | 11 | -1 | 17 | -9 | 44 | 2.216486 |  |  |
| hsa-miR-1244 | 0 | 11 | -1 | 17 | -9 | 44 | 2.216486 |  |  |
| hsa-miR-520a-3p | -1 | 12 | 0 | 16 | -9 | 44 | 2.235507 |  |  |
| hsa-miR-548c-5p | -1 | 12 | 0 | 16 | -9 | 44 | 2.235507 |  |  |
| hsa-miR-302a | -1 | 12 | 0 | 16 | -9 | 44 | 2.235507 |  |  |
| hsa-miR-320b | -1 | 12 | 0 | 16 | -9 | 44 | 2.235507 |  |  |
| hsa-miR-518e | 0 | 11 | -2 | 18 | -8 | 43 | 2.23913 |  |  |
| hsa-miR-100 | 0 | 11 | -2 | 18 | -8 | 43 | 2.23913 |  |  |
| hsa-miR-183 | -1 | 12 | -2 | 18 | -5 | 40 | 2.23913 |  |  |
| hsa-miR-512-5p | 0 | 11 | -3 | 19 | -6 | 41 | 2.240942 |  |  |
| hsa-miR-127-3p | -1 | 12 | -8 | 24 | 6 | 29 | 2.270833 |  |  |
| hsa-miR-1260 | -3 | 14 | 1 | 15 | -7 | 42 | 2.275362 |  |  |
| hsa-miR-28-5p | -1 | 12 | 0 | 16 | -11 | 46 | 2.277174 |  |  |
| hsa-miR-548b-5p | -2 | 13 | -2 | 18 | -4 | 39 | 2.280797 |  |  |
| hsa-miR-518f | 0 | 11 | -2 | 18 | -10 | 45 | 2.280797 |  |  |
| hsa-miR-1276 | -3 | 14 | 1 | 15 | -8 | 43 | 2.296196 |  |  |
| hsa-miR-383 | -7 | 17 | 0 | 16 | 3 | 32 | 2.298007 |  |  |
| hsa-miR-520b | -1 | 12 | -3 | 19 | -6 | 41 | 2.303442 |  |  |
| hsa-miR-873 | -2 | 13 | -5 | 21 | 1 | 34 | 2.307065 |  |  |
| hsa-miR-517b | 0 | 11 | -5 | 21 | -5 | 40 | 2.307065 |  |  |
| hsa-miR-1253 | -2 | 13 | 1 | 15 | -12 | 47 | 2.317029 |  |  |
| hsa-miR-219-2-3p | 0 | 11 | -2 | 18 | -12 | 47 | 2.322464 |  |  |
| hsa-miR-1270 | -2 | 13 | -2 | 18 | -7 | 42 | 2.343297 |  |  |
| hsa-miR-520c-3p | -1 | 12 | -5 | 21 | -5 | 40 | 2.369565 |  |  |
| hsa-miR-1290 | -5 | 16 | 0 | 16 | -4 | 39 | 2.381341 |  |  |
| hsa-miR-1303 | -5 | 16 | 0 | 16 | -4 | 39 | 2.381341 |  |  |
| hsa-miR-372 | -1 | 12 | -3 | 19 | -11 | 46 | 2.407609 | , |  |
| hsa-miR-373 | -2 | 13 | -1 | 17 | -16 | 48 | 2.424819 | ,,, |  |
| hsa-miR-520d-3p | -3 | 14 | 0 | 16 | -17 | 49 | 2.464674 |  |  |
| hsa-miR-936 | -4 | 15 | -4 | 20 | -9 | 44 | 2.59692 |  |  |

**Table S2.** Prediction of 3'UTR mutation effect on miRNAs binding. miRNAs were ranked according to their differential binding to the mutated 3'UTRs by a consensus prediction of the three prediction programs. Top ranked miRNAs are predicted to bind better to the 3'UTR wild type sequences. For the top/bottom 20 miRNAs, a survey of the reported effect in cancer cells is given.

**Reference**

Foley NH, Bray IM, Tivnan A, Bryan K, Murphy DM, Buckley PG, Ryan J, O'Meara A, O'Sullivan M, Stallings RL (2010) MicroRNA-184 inhibits neuroblastoma cell survival through targeting the serine/threonine kinase AKT2. *Mol Cancer* **9:** 83

Greenberg E, Hershkovitz L, Itzhaki O, Hajdu S, Nemlich Y, Ortenberg R, Gefen N, Edry L, Modai S, Keisari Y, Besser MJ, Schachter J, Shomron N, Markel G (2011) Regulation of Cancer Aggressive Features in Melanoma Cells by MicroRNAs. *PLoS One* **6:** e18936

Guo J, Miao Y, Xiao B, Huan R, Jiang Z, Meng D, Wang Y (2009) Differential expression of microRNA species in human gastric cancer versus non-tumorous tissues. *J Gastroenterol Hepatol* **24:** 652-657

Huang Q, Gumireddy K, Schrier M, le Sage C, Nagel R, Nair S, Egan DA, Li A, Huang G, Klein-Szanto AJ, Gimotty PA, Katsaros D, Coukos G, Zhang L, Pure E, Agami R (2008) The microRNAs miR-373 and miR-520c promote tumour invasion and metastasis. *Nat Cell Biol* **10:** 202-210

Kim KS, Cho WJ, Shin JM, Kim JS, Lee MR, Hong KS, Lee JH, Koo KH, Park JW (2009) miR-372 regulates cell cycle and apoptosis of ags human gastric cancer cell line through direct regulation of LATS2. *Molecules and Cells* **28:** 521-527

Lee KH, Goan YG, Hsiao M, Lee CH, Jian SH, Lin JT, Chen YL, Lu PJ (2009) MicroRNA-373 (miR-373) post-transcriptionally regulates large tumor suppressor, homolog 2 (LATS2) and stimulates proliferation in human esophageal cancer. *Exp Cell Res* **315:** 2529-2538

Li JH, Xiao X, Zhang YN, Wang YM, Feng LM, Wu YM, Zhang YX (2011) MicroRNA miR-886-5p inhibits apoptosis by down-regulating Bax expression in human cervical carcinoma cells. *Gynecol Oncol* **120:** 145-151

Li L, Zhang ZM, Liu Y, Wei MH, Xue LY, Zou SM, Di XB, Han NJ, Zhang KT, Xu ZG, Gao YN (2010) [DNA microarrays-based microRNA expression profiles derived from formalin-fixed paraffin-embedded tissue blocks of squammous cell carcinoma of larynx]. *Zhonghua Bing Li Xue Za Zhi* **39:** 391-395

Malzkorn B, Wolter M, Liesenberg F, Grzendowski M, Stuhler K, Meyer HE, Reifenberger G (2010) Identification and functional characterization of microRNAs involved in the malignant progression of gliomas. *Brain Pathol* **20:** 539-550

Nymark P, Guled M, Borze I, Faisal A, Lahti L, Salmenkivi K, Kettunen E, Anttila S, Knuutila S (2011) Integrative analysis of microRNA, mRNA and aCGH data reveals asbestos- and histology-related changes in lung cancer. *Genes Chromosomes Cancer* **50:** 585-597

Pan J, Hu H, Zhou Z, Sun L, Peng L, Yu L, Liu J, Yang Z, Ran Y (2010) Tumor-suppressive mir-663 gene induces mitotic catastrophe growth arrest in human gastric cancer cells. *Oncol Rep* **24:** 105-112

Shu YQ, Gao W, Shen H, Liu LX, Xu JA, Xu J (2011) MiR-21 overexpression in human primary squamous cell lung carcinoma is associated with poor patient prognosis. *Journal of Cancer Research and Clinical Oncology* **137:** 557-566

Tian RQ, Wang XH, Hou LJ, Jia WH, Yang Q, Li YX, Liu M, Li X, Tang H (2011) MicroRNA-372 is down-regulated and targets cyclin-dependent kinase 2 (CDK2) and cyclin A1 in human cervical cancer, which may contribute to tumorigenesis. *J Biol Chem*

Tili E, Michaille JJ, Alder H, Volinia S, Delmas D, Latruffe N, Croce CM (2010) Resveratrol modulates the levels of microRNAs targeting genes encoding tumor-suppressors and effectors of TGFbeta signaling pathway in SW480 cells. *Biochem Pharmacol* **80:** 2057-2065

Tsai KW, Wu CW, Hu LY, Li SC, Liao YL, Lai CH, Kao HW, Fang WL, Huang KH, Chan WC, Lin WC (2011) Epigenetic regulation of miR-34b and miR-129 expression in gastric cancer. *Int J Cancer*

Voorhoeve PM, le Sage C, Schrier M, Gillis AJM, Stoop H, Nagel R, Liu YP, van Duijse J, Drost J, Griekspoor A, Zlotorynski E, Yabuta N, De Vita G, Nojima H, Looijenga LHJ, Agami R (2006) A genetic screen implicates miRNA-372 and miRNA-373 as oncogenes in testicular germ cell tumors. *Cell* **124:** 1169-1181

Yang K, Handorean AM, Iczkowski KA (2009) MicroRNAs 373 and 520c are downregulated in prostate cancer, suppress CD44 translation and enhance invasion of prostate cancer cells in vitro. *Int J Clin Exp Pathol* **2:** 361-369

Yao Y, Suo AL, Li ZF, Liu LY, Tian T, Ni L, Zhang WG, Nan KJ, Song TS, Huang C (2009) MicroRNA profiling of human gastric cancer. *Mol Med Report* **2:** 963-970

Yuen APW, Wong TS, Liu XB, Wong BYH, Ng RWM, Wei WI (2008) Mature miR-184 as potential oncogenic microRNA of squamous cell carcinoma of tongue. *Clinical Cancer Research* **14:** 2588-2592

Zhang C, Wang C, Chen X, Yang C, Li K, Wang J, Dai J, Hu Z, Zhou X, Chen L, Zhang Y, Li Y, Qiu H, Xing J, Liang Z, Ren B, Zen K, Zhang CY (2010) Expression profile of microRNAs in serum: a fingerprint for esophageal squamous cell carcinoma. *Clin Chem* **56:** 1871-1879
