# Supplementary material for: Mutagen-Specific Mutation Signature Determines Global microRNA Binding
Source: PLoS One. 2011 Nov 9;6(11):e27400. doi: 10.1371/journal.pone.0027400 (PMC3212558; doi:10.1371/journal.pone.0027400)
Supplement: Table S3 — Functional enrichment analysis. Highlighted biological processes of table S2 top/bottom ranked 20 miRNAs. (DOC) [file pone.0027400.s003.doc]

**Table S3.** Functional enrichment analysis.

| **First 20 miRNAs** | | | **Last 20 miRNAs** | | |
| --- | --- | --- | --- | --- | --- |
| **Type** | **P-value** | **Targets** | **Type** | **P-value** | **Targets** |
| **Biological Process** | | | **Biological Process** | | |
| cell projection organization | <0.001 | 91/776 | apoptosis | 0.002 | 123/1454 |
| cell part morphogenesis | <0.001 | 70/570 | programmed cell death | 0.003 | 123/1468 |
| cell projection morphogenesis | <0.001 | 69/564 | MAPKKK cascade | 0.007 | 46/405 |
| cell morphogenesis involved in neuron differentiation | <0.001 | 65/525 | regulation of programmed cell death | 0.009 | 100/1159 |
| actin cytoskeleton organization | <0.001 | 48/341 | regulation of apoptosis | 0.010 | 99/1147 |
| neuron projection morphogenesis | <0.001 | 64/523 | regulation of cell death | 0.012 | 101/1181 |
| axonogenesis | <0.001 | 60/482 | cellular response to stress | 0.046 | 80/907 |
| cytoskeleton organization | <0.001 | 73/641 |  |  |  |
| neuron projection development | <0.001 | 71/625 |  |  |  |
| neuron differentiation | <0.001 | 92/894 |  |  |  |
| cell morphogenesis involved in differentiation | 0.001 | 68/606 |  |  |  |
| actin filament-based process | 0.001 | 49/383 |  |  |  |
| neuron development | 0.001 | 76/715 |  |  |  |
| cell morphogenesis | 0.001 | 74/698 |  |  |  |
| generation of neurons | 0.002 | 94/960 |  |  |  |
| cellular component morphogenesis | 0.002 | 77/739 |  |  |  |
| neurogenesis | 0.002 | 98/1016 |  |  |  |
| regulation of cell projection organization | 0.003 | 30/191 |  |  |  |
| regulation of cellular component organization | 0.003 | 86/868 |  |  |  |
| locomotion | 0.003 | 101/1071 |  |  |  |
| axon guidance | 0.005 | 44/350 |  |  |  |
| positive regulation of cell projection organization | 0.005 | 20/101 |  |  |  |
| vesicle-mediated transport | 0.012 | 81/831 |  |  |  |
| positive regulation of gene expression | 0.021 | 87/924 |  |  |  |
| protein localization | 0.029 | 110/1252 |  |  |  |
| cell development | 0.036 | 109/1244 |  |  |  |
| regulation of cell differentiation | 0.037 | 74/762 |  |  |  |

**Table S3.** Functional enrichment analysis. Highlighted biological processes of supplementary table 2 top/bottom ranked 20 miRNAs.
