# Supplementary material for: Mutagen-Specific Mutation Signature Determines Global microRNA Binding
Source: PLoS One. 2011 Nov 9;6(11):e27400. doi: 10.1371/journal.pone.0027400 (PMC3212558; doi:10.1371/journal.pone.0027400)
Supplement: Figure S1 — Nucleotide composition plots. A. 3′UTR composition of mutated genes in melanoma, lung cancer and AML. B. microRNAs compositions. T represent U. (DOC) [file pone.0027400.s004.doc]

| **A**  Melanoma  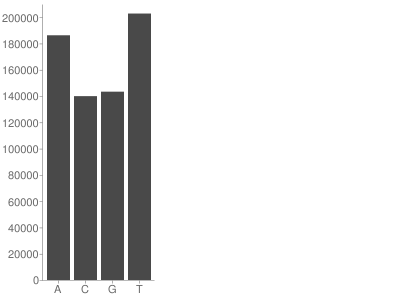 | Lung  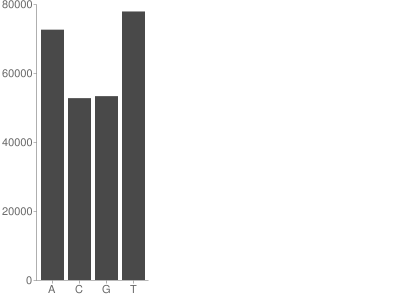 | AML  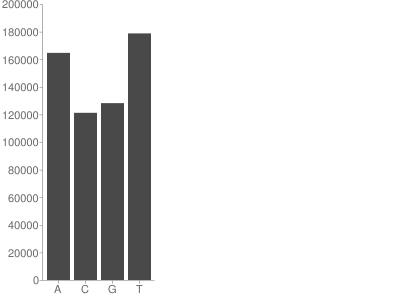 |
| --- | --- | --- |
| **B**  miRNA composition  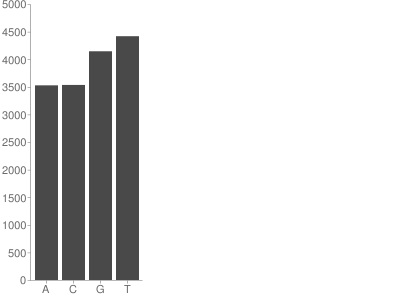 | miRNA seed composition  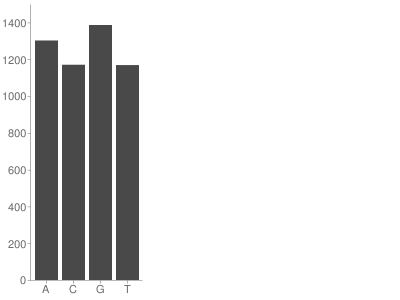 |  |

**Figure S1**

**Figure S1.** Nucleotide composition plots. **A.** 3'UTR composition of mutated genes in melanoma, lung cancer and AML. **B.** microRNAs compositions. T represent U.
